# Robotic *vs*. Retropubic radical prostatectomy in prostate cancer: A systematic review and a meta-analysis update

**DOI:** 10.18632/oncotarget.13332

**Published:** 2016-11-12

**Authors:** Kun Tang, Kehua Jiang, Hongbo Chen, Zhiqiang Chen, Hua Xu, Zhangqun Ye

**Affiliations:** ^1^ Department of Urology, Institute of Urology, Tongji Hospital, Tongji Medical College, Huazhong University of Science and Technology, Wuhan, China; ^2^ Department of Urology, The Central Hospital of Enshi Autonomous Prefecture, Enshi, China

**Keywords:** robotic-assisted radical prostatectomy, retropubic radical prostatectomy, prostate cancer, meta-analysis, update

## Abstract

**CONTEXT:**

The safety and feasibility of robotic-assisted radical prostatectomy (RARP) compared with retropubic radical prostatectomy(RRP) is debated. Recently, a number of large-scale and high-quality studies have been conducted.

**OBJECTIVE:**

To obtain a more valid assessment, we update the meta-analysis of RARP compared with RRP to assessed its safety and feasibility in treatment of prostate cancer.

**METHODS:**

A systematic search of Medline, Embase, Pubmed, and the Cochrane Library was performed to identify studies that compared RARP with RRP. Outcomes of interest included perioperative, pathologic variables and complications.

**RESULTS:**

78 studies assessing RARP vs. RRP were included for meta-analysis. Although patients underwent RRP have shorter operative time than RARP (WMD: 39.85 minutes; *P* < 0.001), patients underwent RARP have less intraoperative blood loss (WMD = -507.67ml; *P* < 0.001), lower blood transfusion rates (OR = 0.13; *P* < 0.001), shorter time to remove catheter (WMD = -3.04day; *P* < 0.001), shorter hospital stay (WMD = -1.62day; *P* < 0.001), lower PSM rates (OR:0.88; *P* = 0.04), fewer positive lymph nodes (OR:0.45;*P* < 0.001), fewer overall complications (OR:0.43; *P* < 0.001), higher 3- and 12-mo potent recovery rate (OR:3.19;*P* = 0.02; OR:2.37; *P* = 0.005, respectively), and lower readmission rate (OR:0.70, *P* = 0.03). The biochemical recurrence free survival of RARP is better than RRP (OR:1.33, *P* = 0.04). All the other calculated results are similar between the two groups.

**CONCLUSIONS:**

Our results indicate that RARP appears to be safe and effective to its counterpart RRP in selected patients.

## INTRODUCTION

Prostate cancer (PCa) is the most common cancer in the worldwide and its morbidity,mortality is the first and second common cancer in men, respectively [1]. RP is the standard therapy for patients with localized PCa [2]. However, open retropubic radical prostatectomy (RRP) is associated with higher overall complications, including estimated blood loss (EBL), wound infections. With the development of surgical techniques, laparoscopic techniques and robot assisted surgeries have become a very popular procedure for the management of urological disease throughout the world [3]. Compared with RRP, the advantages of laparoscopic radical prostatectomy (LRP) are less EBL, fewer complications, better cosmetic effect and shorter hospital stay [4]. The disadvantages of LRP is lack of 3D visualization and poor ergonomics.

As alternatives to open surgery, RARP has became a predominant procedure for the treatment the localized prostate cancer in the world [5]. Assessing of the robotic surgery by expert indicate better ergonomics and quicker learning curve, but its shortage is high cost of the robotic surgery system.

In recent years, many experts have reported on comparative study of RARP and open RRP. And some meta-analysis were performed to evaluate the advantages and disadvantages of two approaches, including perioperative outcomes, oncologic outcomes [5]. Their early experience showed that the outcomes of this approach with fewer overall complications, quicker convalescence, and lower EBL and transfusion [5–7]. However, the outcomes of RARP compared with RRP have not been fully evaluated, and no conclusive results are available. Therefore, a systematic review and meta-analysis of the included published studies was performed to compare RARP with RRP.

## RESULTS

### Characteristics of eligible studies

**Figure 1:**
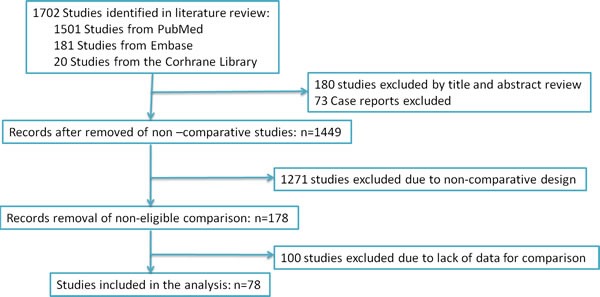
PRISMA diagram The search strategy and number of studies identified for inclusion in this meta-analysis.

According to search strategy, the included 78 studies [4, 8–85] assessing RARP *vs*. RRP met the inclusion criteria and were applied to perform this meta-analysis (Figure 1). Those studies include forty-three retrospective and thirty-five prospective studies and were listed in Table 1.

**Table 1:** Characteristics of included studies

First author, year	Country	Study interval	Design	LOE	No.of patients RARP/RRP	Matching/ comparable*	Quality score^Δ^
Ahlering, 2004	USA	2001-2002	Prospective	3b	60/60	1, 2, 3, 4	*****
Bae, 2012	Korea	2008-2011	Retrospective	3b	111/70	1, 2, 3, 4, 5, 6	*******
Ball, 2006	USA	2000-2005	Prospective	3b	82/135	1, 3, 5, 6	*****
Barocas, 2010	USA	2003-2008	Prospective	3b	1413/491	1, 3, 7	*****
Bolenz, 2010	USA	2003-2008	Retrospective	3b	262/161	1, 2, 3, 4, 6	******
Breyer, 2010	USA	2002-2008	Prospective	3b	293/695	1, 3, 4, 5, 6, 7	*******
Carlsson, 2010	Sweden	2002-2007	Prospective	3b	1253/485	1, 3, 4, 5,	*****
Chan, 2008	USA	2003-2006	Retrospective	3b	660/340	1, 3, 5, 6	*****
Chino, 2009	USA	2003-2007	Retrospective	3b	368/536	1, 3, 5, 6	*****
Choi, 2012	Korea	2007-2011	Retrospective	3b	354/247	1, 3, 5	****
Choo, 2013	Korea	2003-2010	Prospective	3b	77/176	1, 2, 3, 4, 5, 6, 7	*******
Chung, 2012	Taiwan	2006-2009	Retrospective	4	274/1773	1, 7	****
D'Alonzo, 2009	USA	2003-2006	Retrospective	3b	256/280	1, 2, 3, 4, 7	******
Di Pierro, 2011	Switzerland	2007-2009	Prospective	3b	75/75	1, 3, 5, 6, 7	*****
Doumerc, 2010	France	2006-2008	Prospective	3b	212/502	1, 3, 4, 5, 6, 7	*******
Drouin, 2009	France	2000-2004	Retrospective	3b	71/83	1, 2, 3, 5, 6, 7	*******
Farnham, 2006	USA	2003-2004	Prospective	3b	176/103	1, 3, 4, 6	*****
Ficarra, 2009	Italy	2006-2007	Prospective	3b	103/105	1, 2, 3, 4, 5, 6, 7	*******
Fracalanza, 2008	Italy	2006	Prospective	3b	35/26	1, 2, 3, 4, 6	******
Forehner, 2013	Germany	2007-2011	Prospective	3b	252/1925	1, 3, 6, 7	*****
Hong, 2010	Korea	2007	Retrospective	4	26/25	1, 2, 7	****
Park, 2014	Korea	2007-2012	Retrospective	3b	730/277	1, 2, 3, 4, 5, 6, 7	*******
Busch, 2015	Germany	NA	Prospective	3b	194/194	1, 3, 5, 6	*****
Kim, 2011	Korea	2007-2010	Prospective	3b	528/235	1, 2, 3, 5, 6, 7	******
Kordan, 2010	USA	2003-2006	Prospective	3b	830/414	1, 2, 3, 5, 6	*****
Krambeck, 2008	USA	2002-2005	Prospective	3b	294/588	1, 2, 3, 5, 6, 7	*******
Laurila, 2009	USA	2006	Retrospective	3b	94/98	1, 3, 5, 6	*****
Lo, 2010	HongKong	2006-2007	Retrospective	3b	20/20	1, 3, 5, 6, 7	*****
Magheli, 2011	USA	2000-2008	Prospective	3b	522/522	1, 3, 4, 5, 6, 7	*****
Malcolm, 2010	USA	2000-2008	Retrospective	3b	477/135	1, 3, 5, 6, 7	*****
Menon, 2002	France	2001	Prospective	3b	30/30	1, 3, 4, 5, 6, 7	*******
Miller, 2007	USA	2002-2006	Prospective	4	42/120	1	****
Minniti, 2011	Italy	2007-2008	Prospective	3b	22/93	1, 2, 3, 5	****
Nelson, 2007	USA	2003-2006	Prospective	3b	629/374	1, 3, 6	*****
OU, 2009	Taiwan	2004-2007	Retrospective	3b	30/30	1, 2, 3, 4, 5, 6	*******
Pilecki, 2014	USA	2011	Retrospective	4	4374/1097	1, 2	****
Rocco, 2009	Italy	2004-2007	Prospective	3b	120/240	1, 3, 5, 6, 7	******
Ryu, 2013	Korea	2007-2012	Prospective	4	524/341	1, 2, 3, 4	*****
Schroeck, 2008	USA	2003-2007	Retrospective	3b	362/435	1, 2, 3, 4, 5, 6, 7	*******
Shapiro, 2014	USA	2000-2010	Retrospective	3b	108/229	1, 3, 5, 6, 7	*****
Silberstein, 2012	USA	2010	Retrospective	4	126/126	1, 3, 5, 6	*****
Smith, 2007	USA	2002-2006	Retrospective	3b	200/200	1, 2, 3, 4, 5, 6	*******
Son, 2013	Korea	2006-2009	Retrospective	3b	146/112	1, 2, 3, 4, 6, 7	******
Stranne, 2010	Sweden	2002-2006	Retrospective	3b	946/465	1, 2, 3, 4, 5, 6, 7	*******
Sugihara, 2014	Japan	2012-2013	Retrospective	3b	2126/7202	1, 2, 5,	****
Tewari, 2003	USA	1999-2002	Prospective	3b	200/100	1, 2, 3, 4, 5, 6, 7	*******
Truesdale, 2010	USA	2005-2009	Retrospective	3b	99/217	1, 2, 3, 5, 6	******
Vora, 2013	USA	1997-2010	Retrospective	3b	140/95	1, 3, 5, 6, 7	*****
White, 2009	USA	2005-2008	Retrospective	3b	50/50	1, 3, 5, 6	*****
Williams, 2010	USA	2005-2008	Retrospective	4	604/346	1, 3, 5, 6	*****
Wood, 2007	USA	2003-2005	Prospective	4	165/152	1, 3, 7	*****
Yi, 2010	Korea	2006-2009	Retrospective	3b	153/641	1, 2, 3, 6, 7	*****
Rush, 2015	Canada	2009-2012	Retrospective	3b	331/643	1, 2, 3, 4, 7	*****
Ong, 2015	Australian	2009-2012	Prospective	3b	885/1117	1, 3, 5, 6, 7	******
Porcaro, 2015	Italy	2013	Retrospective	4	108/43	1, 2, 3, 4, 5, 6, 7	*******
O'Neil, 2015	USA	2011-2012	Prospective	3b	933/1505	1, 3, 6, 7	****
Niklas, 2015	Germany	2003-2010	Retrospective	3b	932/499	1, 2, 3, 4, 5, 6, 7	*******
Haglind, 2015	Sweden	2008-2011	Prospective	3b	1847/778	1, 2, 3, 5, 6	******
Gagnon, 2014	Canada	NA	Retrospective	3b	200/200	1, 2, 3, 4, 5, 6, 7	******
Davison, 2014	Canada	2007-2009	Prospective	3b	78/73	1, 3, 5	*****
Akand, 2015	Turkey	1999-2012	Retrospective	4	79/50	1, 2, 3, 4, 5, 7	******
Korets, 2014	USA	2007-2012	Retrospective	3b	12746/3398	1, 2, 7	*****
Wallerstedt, 2015	Sweden	NA	Prospective	3b	1847/778	1, 2, 3, 5, 6, 7	******
Hu, 2015	USA	2004-2009	Retrospective	3b	5524/7878	1, 2, 3, 5, 6, 7	******
Davis, 2014	USA	2004-2010	Prospective	3b	27348/13840	1, 7	****
Rithch, 2014	USA	2003-2009	Retrospective	3b	742/237	1, 2, 3, 5, 6, 7	******
Gandaglia, 2014	USA	2008-2009	Retrospective	3b	3476/2439	1, 3, 5, 6, 7	*****
Koo, 2014	Korea	1992-2008	Retrospective	3b	175/175	1, 3, 5, 6, 7	******
Busch, 2014	Germany	NA	Retrospective	3b	110/110	1, 2, 3, 4, 5, 6, 7	*******
Alemozaffar, 2015	USA	2000-2010	Prospective	3b	282/621	1, 2, 3, 4, 5, 6, 7	*******
Harty, 2013	USA	2000-2010	Prospective	3b	152/153	1, 3, 4, 5, 6, 7	*******
Silberstein, 2013	USA	2007-2010	Retrospective	3b	493/961	1, 3, 5, 7	*****
Ludovico, 2013	Italy	2004-2008	Retrospective	3b	82/48	1, 3, 5, 6, 7	******
Musch, 2013	Germany	2009-2010	Retrospective	3b	105/105	1, 2, 3, 4, 5, 6, 7	******
Hall, 2014	Australia	2007-2009	Retrospective	3b	100/100	1, 3, 6	*****
Geraerts, 2013	Belgium	2009-2011	Prospective	3b	64/116	1, 2, 7	*****
Drouin, 2014	France	2007-2010	Prospective	3b	73/44	1, 3, 5, 6, 7	******
Pierorazio, 2013	USA	2002-2011	Retrospective	3b	105/743	1, 2, 3, 4, 5, 6, 7	*******

RARP=robot-assisted radical prostatectomy; RRP= retropubic radical prostatectomy; NA= data not available; LOE= level of evidence.

*:Matching/comparable variable: 1=age, 2=BMI, 3=PSA, 4=prostate size, 5=clinical stage, 6= biospy Gleason score, 7=follow up

Δ:based on Newcastle-Ottawa Scale.

Quality of the studies and level of evidence (Table 1)In this meat-analysis, the Newcastle-Ottawa Scale quality assessment method of the observational studies [86], and the US Preventive Services Task Force grading system [87] were applied to evaluate the quality of included studies. Twenty studies scored seven stars and were evaluated as the high quality studies. Additionally, The clinical variables of RARP and RRP were extracted independently from included literatures (Table 1).

### Description of included studies and patients Demographics (Table 2)

**Table 2:** Overall analysis of demographic and clinical characteristics compared RARP with RRP

Outcomes of interest	No. of studies	No. of patients RARP/RRP	OR/WMD(95% CI )	*p*-value	Study heterogeneity			
					Chi^2^	df	*I*^2^	*p*-value
Age(year)	33	41866/227181	-1.00[-1.56,-0.44]	**<0.001**	1260.51	32	97%	**<0.001**
BMI(kg/m^2^)	17	9365/4690	-0.10[-0.39,0.20]	0.52	87.93	16	82%	**<0.001**
Pre-PSA(ng/ml)	23	6161/5250	-0.93[-1.47,-0.40]	**<0.001**	234.69	22	91%	**<0.001**
Prostate volume(ml)	12	3995/3288	2.35[-0.92,5.61]	0.16	136.49	11	92%	**<0.001**

RARP=robot-assisted radical prostatectomy; RRP=retropubic radical prostatectomy; OR = odds ratio; WMD = weighted mean difference; CI = confidence interval; BMI = body mass index.

Patients underwent RARP are younger (WMD = -1.00 years; 95% CI: -1.56 to -0.44; *P* < 0.001) (Figure S1), and have the lower level of pre-PSA (OR = -0.93; 95% CI: -1.47 to -0.40; *P* < 0.001) (Figure S2). But there is no significant difference on BMI (OR = -0.10; 95% CI: -0.39 to 0.20;*P* = 0.20) (Figure S3), and prostate volume (WMD = 2.35ml; 95% CI: -0.92 to 5.61; *P* = 0.16) (Figure S4) between the RARP and RRP group. (Table 2).

### Outcomes of perioperative variables (Table 3)

**Table 3:** Overall analysis of perioperative outcomes comparing RARP with RRP

Outcome of interest	No. of studies	No.of patients RARP/RRP	OR/WMD(95%CI) ^†^	*p*-value	Study heterogeneity			
					Chi^2^	df	*I*^2^	*p*-value
Operation time, min	18	36296/17965	39.85[20.^95^,^58^.^75^] ^†^	**<0.001**	2130.01	17	99%	**<0.001**
Estimated blood loss, ml	13	3446/2791	-507.67[-633.21,-382.12] ^†^	**<0.001**	390.34	12	97%	**<0.001**
Transfusion rate	26	54847/32967	0.13[0.08,0.21]	**<0.001**	693.85	25	96%	**<0.001**
Remove the catheter, day	5	2135/1264	-3.04[-4.59,-1.49] ^†^	**<0.001**	260.52	4	98%	**<0.001**
Hospital stay, day	11	32196/17106	-1.62[-2.42,-0.82] ^†^	**<0.001**	1517.19	10	99%	**<0.001**

RARP=robot-assisted radical prostatectomy; RRP=retropubic radical prostatectomy; OR = odds ratio; WMD = weighted mean difference; CI = confidence interval.

#### Operating time and estimated blood loss (EBL)

**Figure 2:**
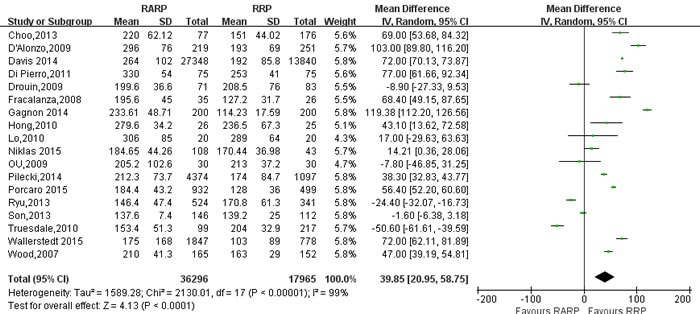
Forest plot and meta-analysis of operating time between RARP and RRP RARP = robot-assisted radical prostatectomy; RRP = retropubic radical prostatectomy.

**Figure 3:**
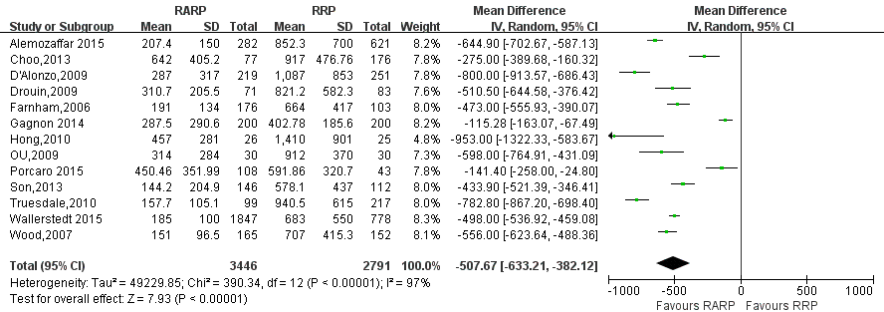
Forest plot and meta-analysis of estimated blood loss between RARP and RRP RARP = robot-assisted radical prostatectomy; RRP = retropubic radical prostatectomy.

With respect to perioperative variables, pooling data of 18 studies [21, 23, 24, 26, 29, 32, 34, 40, 54, 59, 60, 64, 70, 75, 78, 81, 84, 85] involving 54261 participants indicated that RARP has longer operative time than RRP (WMD: 39.85 minutes; 95% CI: 20.95 to 58.75; *P* < 0.001) (Figure 2). Pooling data of 13 studies [10, 21, 23, 29, 30, 34, 40, 60, 70, 75, 78, 84, 85] results showed that RARP has less intraoperative blood loss (WMD = -507.67ml; 95% CI: -633.21 to -382.12; *P* < 0.001) (Figure 3).

#### Transfusion rate and postoperative recovery

**Figure 4:**
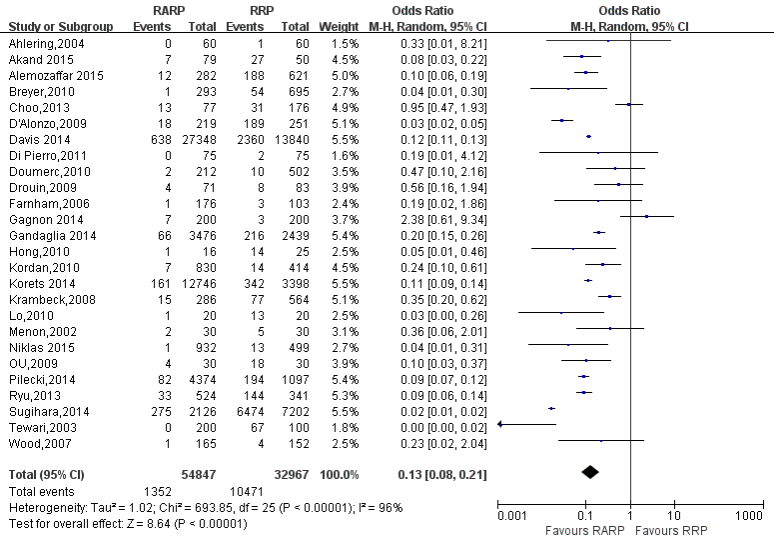
Forest plot and meta-analysis of transfusion rate between RARP and RRP RARP = robot-assisted radical prostatectomy; RRP = retropubic radical prostatectomy.

**Figure 5:**
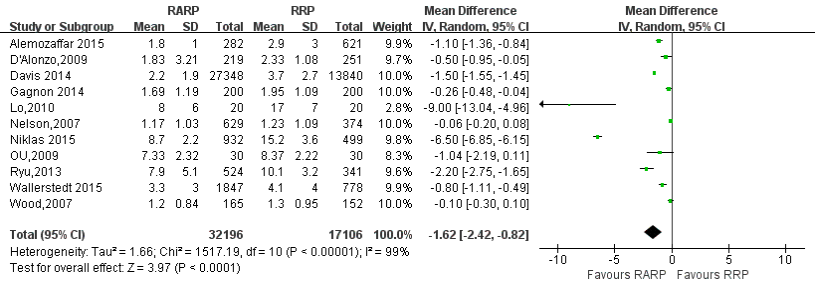
Forest plot and meta-analysis of the length of hospital stay between RARP and RRP RARP = robot-assisted radical prostatectomy; RRP = retropubic radical prostatectomy.

Pooled data from the 26 studies [9, 10, 14, 21, 23, 24, 26, 29, 30, 34, 35, 40, 44-46, 54, 59, 64, 72, 73, 78, 80, 82, 84] reported transfusion rate between RARP and RRP, and the results showed that RARP was associated with lower transfusion rate (OR = 0.13; 95% CI: 0.08 to 0.21;*P* < 0.001) than RRP (Figure 4). Pooling data of 5 studies reported on the time to remove catheter, the forest plot showed that RARP had shorter time to remove catheter than RRP group (WMD = -3.04; 95% CI: -4.59 to -1.49; *P* < 0.001) (Figure S5). And pooling date of 11 studies [10, 23, 24, 34, 53, 54, 64, 75, 78] reported on length of hospital stay (LOS), the forest plot showed that RARP had a shorter LOS than RRP (WMD = -1.62; 95% CI: -2.42 to -0.82; *P* < 0.001) (Figure 5).

### Outcomes of oncological variables

#### pathologic stage and pathologic Gleason score (Table 5)

**Table 4:** Overall analysis of complications comparing RARP and RRP

Outcome of interest	No. of studies	No.of patients RARP/RRP	OR (95%CI)	*p*-value	Study heterogeneity			
					Chi^2^	df	*I*^2^	*p*-value
Overall complications	25	43087/28834	0.43 [0.32, 0.58]	**<0.001**	499.59	24	95%	**<0.001**
Rectal injury	8	3888/8110	0.16[0.07, 0.39]	**<0.001**	5.22	7	0%	0.63
Pulmonary embolism	9	37575/24635	0.47[0.37, 0.59]	**<0.001**	5.04	8	0%	0.75
Wound infections	10	11161/10587	0.23[0.11, 0.46]	**<0.001**	31.49	9	71%	**<0.001**
Bladder neck contracture	4	1993/2409	0.21[0.08,0.60]	**0.003**	8.39	3	64%	**0.04**
UTI	4	6586/2546	0.75[0.37,1.54]	0.44	15.35	3	80%	**0.002**
Urinary retention	3	2042/960	0.63[0.47,0.84]	**0.002**	2.44	2	18%	0.29
Obturator nerve injury	2	1453/585	0.09[0.01,0.75]	**0.03**	0.01	1	0%	0.91
DVT	7	7479/3072	0.40[0.25,0.66]	**<0.001**	10.82	6	45%	0.09
Urinary leakage	8	30940/15631	0.64[0.58,0.70]	**<0.001**	8.87	7	21%	0.26
ileus	8	3412/8501	0.92[0.56,1.51]	0.73	2.20	7	0%	0.95
lymphocele	9	45258/2639	0.52[0.29,0.94]	**0.03**	8.93	8	10%	0.35
Urinary continence-3mo	9	997/941	1.54[0.92,2.58]	0.10	22.06	8	64%	**0.005**
Urinary continence-12mo	9	1565/2179	1.03[0.84,1.27]	0.75	17.41	8	54%	0.03
Potent recovery-3mo	5	1169/820	3.19[1.19,8.56]	**0.02**	51.94	4	92%	**<0.001**
Potent recovery-12mo	7	1395/1574	2.37[1.30,4.33]	**0.005**	55.43	6	89%	**<0.001**
Readmission rate	7	11632/7060	0.83[0.74,0.94]	**0.002**	36.82	6	84%	**<0.001**

RARP=robot-assisted radical prostatectomy; RRP= retropubic radical prostatectomy; OR = odds ratio; WMD = weighted mean difference; CI = confidence interval; UTI=urinary tract infection; DVT=deep venous thrombosis.

**Table 5:** Overall analysis of pathologic and oncological outcomes comparing RARP with RRP

Outcome of interest	No. of studies	No.of patients RARP/RRP	OR/WMD(95%CI)	*p*-value	Study heterogeneity			
					Chi^2^	df	*I*^2^	*p*-value
**Pathologic T stage**								
≤pT2a	13	2147/2174	1.11[0.93,1.31]	0.26	8.84	12	0%	0.72
pT2b	11	1959/2098	1.11[0.93,1.33]	0.25	13.91	10	28%	0.18
≥pT2c	14	2268/2485	0.93[0.76,1.13]	0.44	11.19	13	0%	0.60
**Pathological Gleason score**								
≤6	48	15238/13412	1.04[0.91,1.18]	0.61	224.21	47	79%	<0.001
7	48	15238/13412	1.17[1.04,1.33]	**0.01**	230.23	47	80%	<0.001
≥8	48	15238/13412	0.68[0.60,0.78]	**<0.001**	101.358	47	54%	<0.001
PSM	49	20804/23133	0.88[0.78,1.00]	**0.04**	198.74	48	76%	<0.001
PSM for T2	28	10086/9711	0.77[0.63,0.95]	**0.01**	82.23	27	67%	**<0.001**
PSM for T3	18	2011/2125	1.46[1.27,1.67]	**<0.001**	18.66	17	9%	0.35
Mean lymph node yield	4	837/565	2.85[-0.92,6.63] ^†^	0.14	115.32	3	97%	**<0.001**
Positive lymph node	16	4162/6500	0.45[0.31,0.65]	**<0.001**	32.02	15	53%	0.006
BCR for free survival	10	4342/4176	1.33[1.01,1.76]	**0.04**	39.04	9	77%	**<0.001**

RARP=robot-assisted radical prostatectomy; RRP=retropubic radical prostatectomy; OR = odds ratio; WMD = weighted mean difference; CI = confidence interval; PSM=positive surgical margins; ^†^value of WMD.

**Figure 6:**
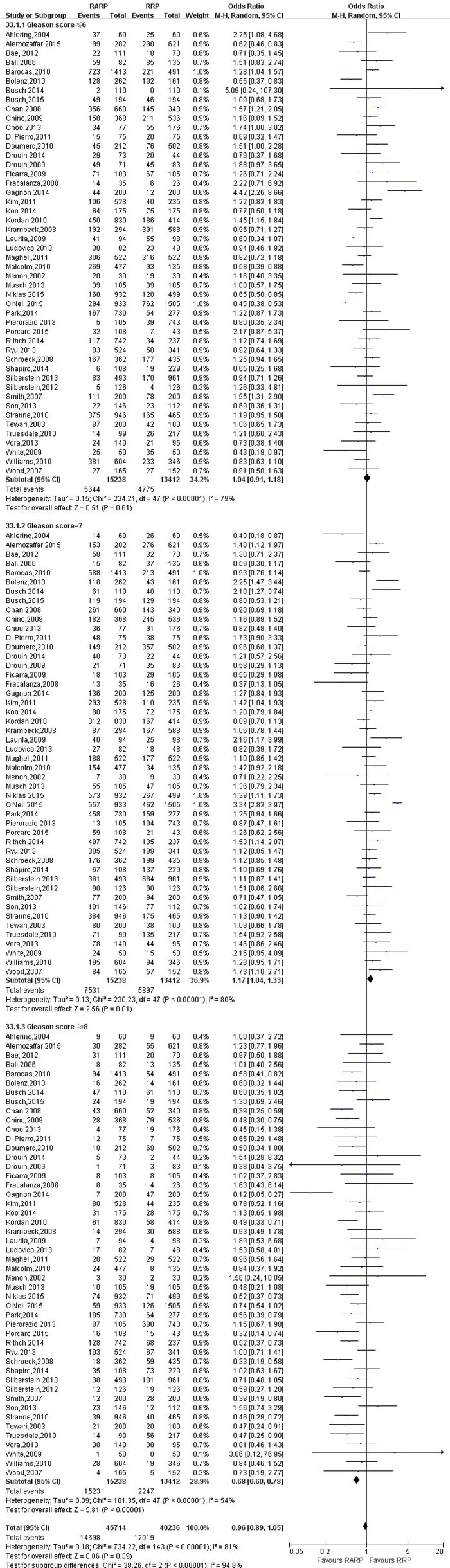
Forest plot and meta-analysis of pathological Gleason Score between RARP and RRP RARP = robot-assisted radical prostatectomy; RRP = retropubic radical prostatectomy.

14 studies [9, 20, 27-29, 32, 46, 48, 66, 70, 73, 76, 77, 80] on ≤pT2a, pT2b, ≥pT2c, 48 studies [8-13, 15, 16, 18, 19, 21, 26-29, 31, 32, 34, 42-44, 46-50, 52, 54, 55, 57, 58, 60, 61, 64-71, 73, 74, 76-78, 82, 85] on pathologic Gleason score (≤6; 7; ≥8) were reported, respectively. The results showed a statistical differences more Gleason score = 7 (OR: 1.17; 95% CI: 1.04 to 1.33; *P* = 0.01; Figure 6) performed RARP and more Gleason score ≥8 (OR: 0.68; 95% CI: 0.60 to 0.78; *P* < 0.001; Figure 6) in RRP. However, there were no statistical differences with respect to Gleason score≤6 (OR: 1.04; 95% CI: 0.91 to 1.18; *P* = 0.61; Figure 6) and pathologic T stage in the two groups (Figure S6,7,8)(Table 5).

#### Positive surgical margins and lymph node yield(Table 5)

**Figure 7:**
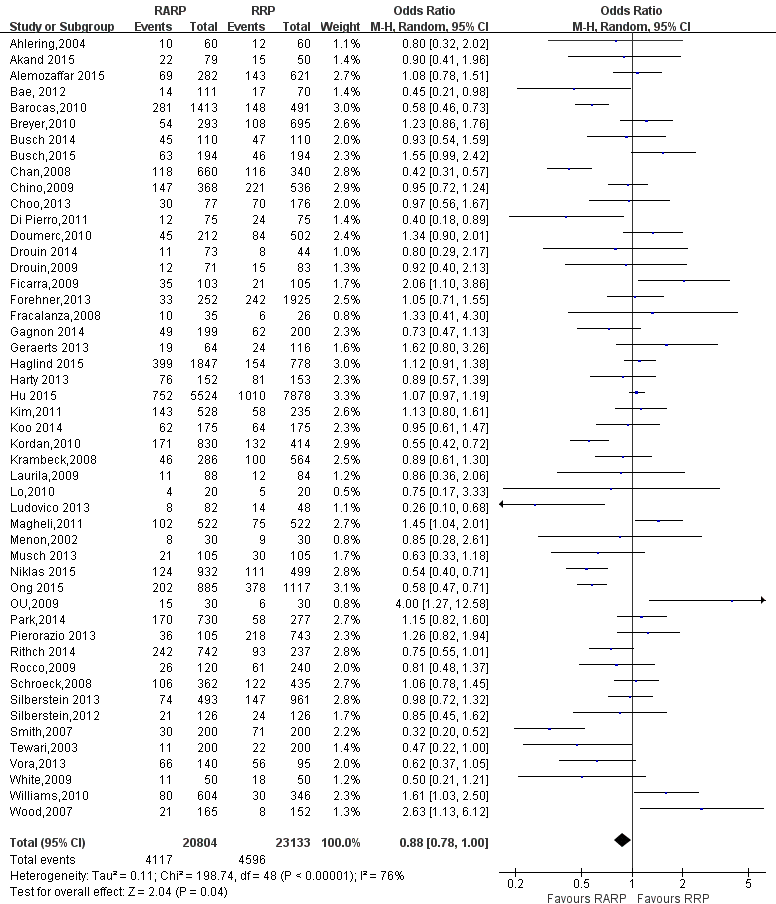
Forest plot and meta-analysis of PSM between RARP and RRP RARP = robot-assisted radical prostatectomy; RRP = retropubic radical prostatectomy.

**Figure 8:**
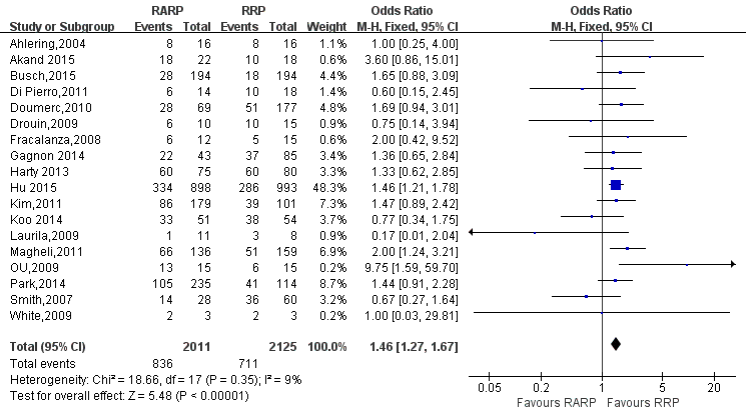
Forest plot and meta-analysis of PSM for pT3 between RARP and RRP RARP = robot-assisted radical prostatectomy; RRP = retropubic radical prostatectomy.

**Figure 9:**
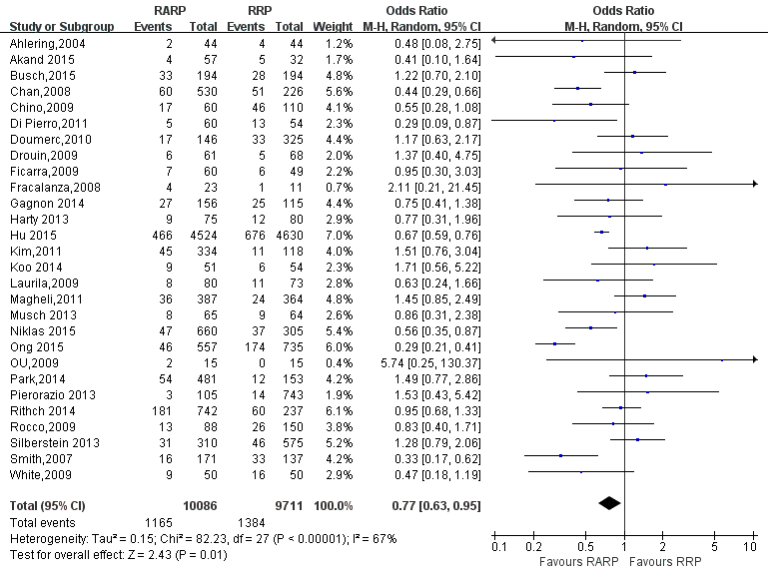
Forest plot and meta-analysis of PSM for pT2 between RARP and RRP RARP = robot-assisted radical prostatectomy; RRP = retropubic radical prostatectomy.

**Figure 10:**
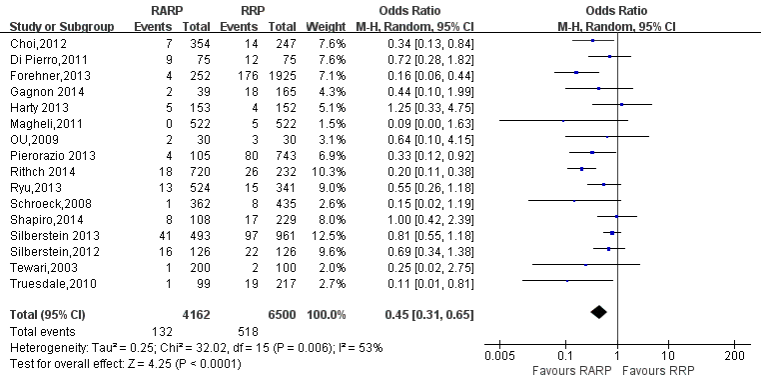
Forest plot and meta-analysis of positive lymph node between RARP and RRP RARP = robot-assisted radical prostatectomy; RRP = retropubic radical prostatectomy.

49 studies [9-12, 14-16, 18, 19, 21, 26-29, 31-34, 36, 37, 39, 41-44, 46-49, 52, 54, 56-58, 61, 62, 65, 67-69, 73, 76-78, 80-82, 84] evaluating RARP and RRP reported positive surgical margins(PSM) rates. The results showed a significant difference with higher PSM rates in RRP group (OR:0.88; 95% CI: 0.78 to 1.00; *P* = 0.04)(Figure 7). PSM rates in pT3 cancers was higher in RARP group (OR:1.46; 95% CI: 1.27 to 1.67; *P* < 0.001) (Figure 8). However, the results showed that PSM rates in pT2 cancers was lower in RARP (OR:0.77; 95% CI: 0.63 to 0.95; *P* = 0.01)(Figure 9). Four studies [20, 43, 60, 73] comparing mean lymph node yield and the results showed that lymph node yield is higher in RARP (WMD: 1.61; 95% CI: 1.18 to 2.05; *P* < 0.001)(Figure S9), and 16 studies [20, 26, 33, 34, 39, 49, 58, 61, 64-68, 73, 84, 85] reported on positive lymph node, There was a statistical differences decreased positive lymph node in RARP than RRP (OR:0.45; 95% CI: 0.31 to 0.65; *P* < 0.001)(Figure 10).

### Outcomes of complications(Table 4)

**Figure 11:**
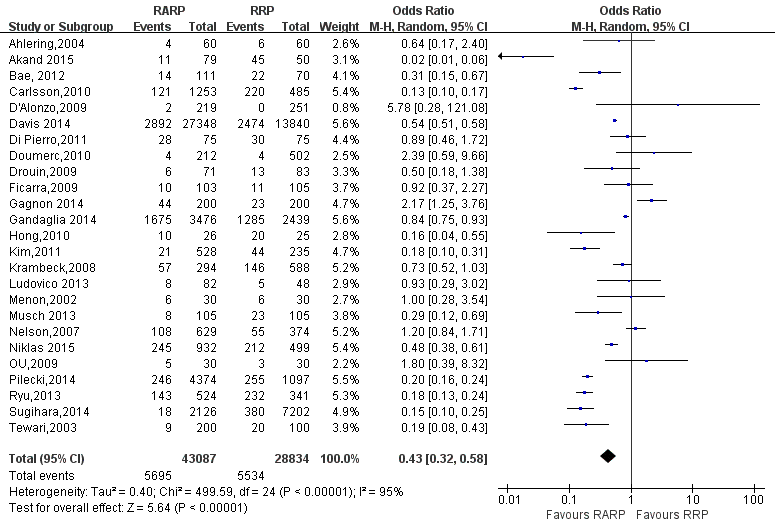
Forest plot and meta-analysis of overall complications between RARP and RRP RARP = robot-assisted radical prostatectomy; RRP = retropubic radical prostatectomy.

Pooling data from 25 studies [9, 11, 17, 23, 24, 26, 27, 29, 31, 34, 35, 40, 42, 46, 48, 52-54, 59, 64, 72, 73, 80, 82, 84] reported on overall complications, RARP had lower overall complications in the RARP than RRP(OR:0.43; 95% CI: 0.32 to 0.58; *P* < 0.001)(Figure 11). Next, a meticulous classification of overall complications showed that RRP had a higher incidence of rectal injury(OR:0.16; 95% CI: 0.07 to 0.39; *P* < 0.001)(Figure S10), pulmonary embolism(OR:0.47; 95% CI: 0.37 to 0.59; *P* < 0.001) (Figure S11), wound infections (OR:0.23; 95% CI: 0.11 to 0.46; *P* < 0.001) (Figure S12), bladder neck contracture(OR: 0.21; 95% CI: 0.08 to 0.60; *P* = 0.003) (Figure S13), urinary retention(OR:0.63; 95% CI: 0.47 to 0.84; *P* = 0.002)(Figure S14), deep venous thrombosis(OR:0.40; 95% CI: 0.25 to 0.66; *P* < 0.001) (Figure S15), urinary leakage(OR: 0.64; 95% CI: 0.58 to 0.70; *P* < 0.001) (Figure S16), lymphocele (OR:0.52; 95% CI: 0.29 to 0.94; *P* = 0.03) (Figure S17), and obturator nerve injury(OR:0.09; 95% CI: 0.01 to 0.75; *P* = 0.03) (Figure S18). There was no statistical differences between two groups in term of urinary tract infections(UTI)(OR:0.75; 95% CI: 0.37 to 1.54; *P* = 0.44)(Figure S19), ileus (OR:0.92; 95% CI: 0.56 to 1.51; *P* = 0.73) (Figure S20).

#### Urinary continence recovery and potent recovery(Table 4)

**Figure 12:**
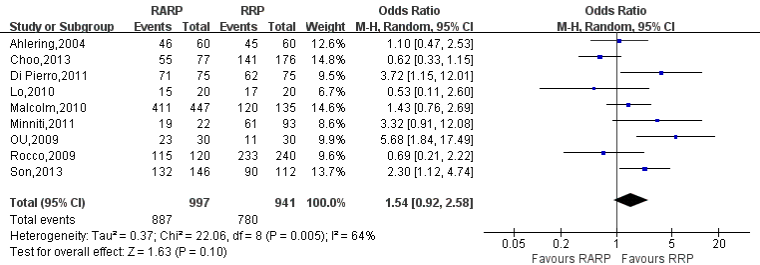
Forest plot and meta-analysis of 3-mo urinary continence rate between RARP and RRP RARP = robot-assisted radical prostatectomy; RRP = retropubic radical prostatectomy.

**Figure 13:**
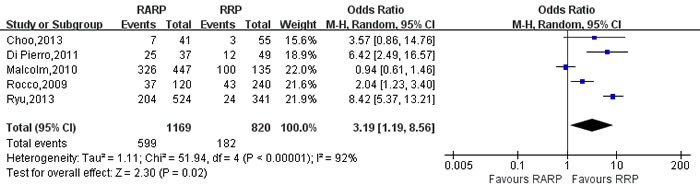
Forest plot and meta-analysis of 3-mo potent recovery rate between RARP and RRP RARP = robot-assisted radical prostatectomy; RRP = retropubic radical prostatectomy.

**Figure 14:**
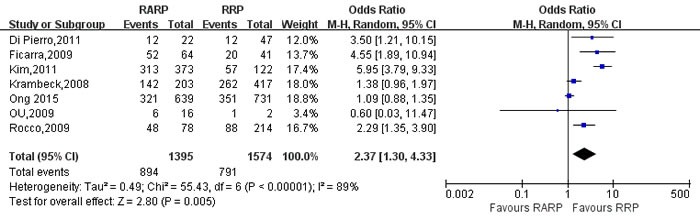
Forest plot and meta-analysis of 12-mo potent recovery rate between RARP and RRP RARP = robot-assisted radical prostatectomy; RRP = retropubic radical prostatectomy.

Pooling data of 9 studies [9, 21, 26, 50, 62, 70, 81, 83, 84] reported on 3-mo and 12-mo urinary continence recovery between two groups. The forest plot showed that there were no statistical differences on the 3-mo and 12-mo urinary continence between two groups (3mo: OR:1.54; 95% CI: 0.92 to 2.58; *P* = 0.10; 12mo: OR:1.03; 95% CI: 0.84 to 1.27; *P* = 0.75,respectively)(Figure 12, Figure S21). And the 3- and 12-mo potent recovery rate of RARP were better than RRP group, respectively (OR:3.19; 95% CI: 1.19 to 8.56; *P* = 0.02; OR: 2.37; 95% CI: 1.30 to 4.33; *P* = 0.005,respectively)(Figures 13,14).

#### Biochemical recurrence free survival and Readmission rate(Table 5)

**Figure 15:**
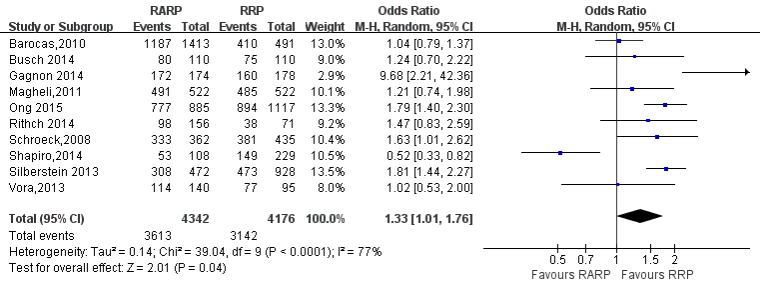
Forest plot and meta-analysis of BCR free survival rate between RARP and RRP RARP = robot-assisted radical prostatectomy; RRP = retropubic radical prostatectomy.

**Figure 16:**
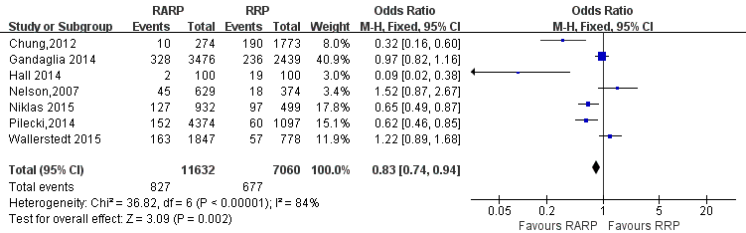
Forest plot and meta-analysis of readmission rate between RARP and RRP RARP = robot-assisted radical prostatectomy; RRP = retropubic radical prostatectomy.

Pooling data from 10 studies [12, 16, 34, 49, 56, 61, 65-67, 74] reported on biochemical recurrence(BCR) free survival, these results showed that RARP had a better BCR free survival than RRP(OR:1.33; 95% CI: 1.01 to 1.76; *P* = 0.04) (Figure 15). Pooling data from 7 studies [22, 35, 38, 53, 54, 59, 75] reported on readmission rate, the forest plot showed that RARP had a lower readmission rate than RRP(OR:0.83; 95% CI: 0.74 to 0.94; *P* = 0.002) (Figure 16).

### Sensitivity analysis

42 qualified studies with patients’ baseline characteristic consistency(age, pre-PSA, BMI, prostate volume, *P* > 0.5) are analyzed by sensitivity analysis (Table 6). Compared with the original analysis, there was no change in the significance of any other outcomes except that readmission rate(*P* = 0.002 *vs* *P* = 0.13), and BCR for free survival(*P =* 0.04 *vs*. *P* = 0.55) were significantly different in sensitivity analysis. The method of sensitivity analysis can reduce the heterogeneity of studies to a certain extent.

**Table 6:** Sensitivity analysis of high quality studies comparing RARP with RRP

Outcome of interest	No. of studies	No.of patients RARP/RRP	OR/WMD(95%CI) ^†^	*p*-value	Study heterogeneity			
					Chi^2^	df	*I*^2^	*p*-value
Operation time, min	10	1523/1435	44.43[8.01,80.84] ^†^	**0.02**	1166.56	9	99%	**<0.0001**
Estimated blood loss, ml	8	1080/1102	-493.41[-672.09,-314.74] ^†^	**<0.001**	217.36	7	97%	**<0.0001**
Transfusion rate	18	16249/7209	0.16[0.09,0.28]	**<0.001**	116.44	17	85%	**<0.0001**
Remove the catheter, day	3	1173/735	-1.78[-2.50,-1.06] ^†^	**<0.001**	19.52	2	90%	**<0.0001**
Hospital stay, day	6	1568/1117	-0.75[-1.26,-0.24] ^†^	**0.004**	75.72	5	93%	**<0.0001**
Overall complications	14	2782/2767	0.50 [0.27, 0.92]	**0.03**	158.13	13	92%	**<0.0001**
Urinary continence-3mo	7	945/818	1.21[0.74,1.98]	0.45	13.33	6	55%	**0.04**
Urinary continence-12mo	4	942/1409	0.97[0.78,1.20]	0.79	10.89	6	45%	0.09
Potent recovery-3mo	4	722/685	4.50[1.91,10.62]	**<0.001**	17.64	3	83%	**<0.001**
Potent recovery-12mo	4	942/1409	1.58[1.05,2.36]	**0.03**	10.33	3	71%	**0.02**
Readmission rate	4	2850/3025	0.53[0.23,1.21]	0.13	24.10	3	88%	**<0.001**
**Pathologic T stage**								
≤pT2a	10	1725/1871	1.02[0.83,1.26]	0.83	7.02	9	0%	0.63
pT2b	9	1675/1821	0.99[0.80,1.21]	0.90	8.07	8	1%	0.43
≥pT2c	12	1979/2212	0.98[0.79,1.21]	0.84	9.48	11	0%	0.58
**Pathological Gleason score**								
≤6	27	5847/6576	0.99[0.87,1.13]	0.88	45.37	26	43%	0.01
7	27	5847/6576	1.14[1.02,1.28]	**0.02**	46.80	26	44%	0.007
≥8	27	5847/6576	0.79[0.67,0.92]	**0.003**	38.31	26	32%	0.06
PSM	39	13992/17806	0.87[0.76,0.99]	**0.04**	123.38	37	70%	**<0.001**
PSM for T2	16	6649/7986	0.71[0.53,0.95]	**0.02**	51.53	15	71%	**<0.001**
PSM for T3	12	1423/1713	1.39[1.19,1.63]	**<0.001**	9.38	11	0%	0.59
Mean lymph node yield	2	375/275	3.77[-5.87,13.41] ^†^	**0.44**	106.54	1	99%	**<0.001**
Positive lymph node	10	2668/3684	0.69[0.52,0.90]	**0.006**	9.31	9	3%	0.41
BCR for free survival	5	1192/1797	1.16[0.71,1.89]	**0.55**	23.76	4	83%	**<0.001**

RARP=robot-assisted radical prostatectomy; RRP=retropubic radical prostatectomy; OR = odds ratio; WMD = weighted mean difference; CI = confidence interval.

## DISCUSSION

The incidence of prostate cancer and its mortality is the first and the second common cancer in man, respectively [1]. Our results indicated that RARP seemed to have an younger age (WMD: -1.00; *P* < 0.001), and to have the lower level of pre-PSA (WMD: -0.93; *P* < 0.001) than RRP group, and that these differences are primarily due to surgeon's preference for surgical modality. Another reason is that the younger is more easier to choose new approach. However, there is no difference on BMI and prostate volume between the two groups. Sensitivity analysis showed that there was no change in the significance of any other outcomes except that readmission rate(*P* = 0.002 *vs* *P* = 0.13) and BCR for free survival(*P =* 0.04 *vs*. *P* = 0.55).It demonstrated that selection bias of demographic and clinical data of patients is small between two groups.

Novara G et al [6] evaluated oncologic outcomes of RARP and RRP, and the results indicated that RARP had less EBL and transfusion rate than RRP. Their results presented similar results and strengthened our results. The other analyzed parameters operative time and complication rate were similar. However, in our meta-analysis, RARP had longer operative time than RRP(WMD:39.85min, *P*<0.001), which likely reflects the early learning curve with RARP. But the learning curve indicated that operative time was decreased with growing operative experience and it won't influenced operative outcomes [88].

With regard to the pathologic outcomes, patients underwent RARP had more pathological Gleason score = 7, less pathological Gleason score ≥8, higher lymph node yield and fewer positive lymph node than RRP. However, the pathological T stage is no significant difference between the RARP and RRP group. LN yield was deemed an indicator of surgical quality by many surgeons [89]. RARP had a higher LN yield than RRP, the reason is that RARP has meticulous dissection with 3D vision and decrease the intraoperative blood loss which made the surgeon have more time and patience to acquiring higher LN yield. Therefore, the oncological outcomes in terms of PSM for T3 is higher in RARP than RRP. With the results that BCR free survival was higher in RARP than RRP. Some studies showed that the predictors of BCR were preoperative PSA. Gleason score, pathological stage, and PSM [49].

The experts suggested that patient outcomes and surgical approach were mainly required to improve for an accurate characterization of complications [90]. In our meta-analysis, Patients underwent RARP had fewer overall complications than RRP. The possible reason may be associated with lower EBL and less transfusion rate in RARP. Then a comprehensive classification of complications indicated that RRP had a higher incidence of rectal injury, pulmonary embolism, wound infections, bladder neck contracture, urinary retention, deep venous thrombosis, urinary leakage, lymphocele, and obturator nerve injury. There were no significant differences with regard to ileus and UTI between two groups.

Ficarra V et al [91] compared RARP with RRP with respect to 12-mo urinary continence. Their results indicated that RARP had a better 12-mo urinary continence recovery than RRP(OR:1.53; *P* = 0.03). However, our results indicated that there were no statistical differences with regard to 3-mo and 12-mo urinary continence in two groups. The urinary continence receiving RP is influenced by preoperative patient characteristics, surgical techniques, and so on. Some studies found that patient age [92, 93], BMI [94], comorbidity index [95], and prostate volume [96, 97] were also the potential predictors of urinary incontinence. Increasing age, higher BMI, and large prostate volume are correlated with high risk of urinary incontinence who underwent RP. However, the 3- and 12-mo potent recovery rate of RARP was also better than RRP group, respectively. Analysis of predictors indicated that peroperative parameters might influence potency results. Relevant predictors included age at surgery, baseline erectile function, and comorbidities [98]. Other authors also confirmed that age and baseline erectile function of patients were affected the potent recovery in nerve-sparing RARP [93, 99].

On the other hand, we found better BCR free survival and lower readmission rate in RARP group in the original analysis. The reason is that meticulous dissection, lower blood loss and complications might provide patients better oncologic prognosis in RARP group. However, we observed no statistical differences between RARP and RRP in sensitivity analysis. Therefore, multicenter, large sample, long follow-up RCTs are required to prove our findings.

Nevertheless, there were several limitations when analyzing and interpreting results in our meta-analysis. The major limitation is lack of well designed prospective, randomized control studies in our meta-analysis. Secondly, there existed heterogeneities of studies, especially in the comparing of the continuous data such as the length of hospital stay, operative time. whereas these parameters were influenced by the heterogeneities of patients’ conditions, surgeon's surgical skills and the sample size of studies. In addition, short follow-up duration may have an influence on the confidence of outcomes. In the future, well-designed, prospective, multicenter randomized control studies are required to help us better demonstrate the advantages as well as drawbacks of this novel approach.

## MATERIALS AND METHODS

### Literature search strategy

To update previous systematic review [5-7, 91, 98, 100, 101], a systematic review of published literature was performed according to the Cochrane Handbook recommendations [102]. No ethic issues get involved in this article. A systematic dissertion was conducted using Medline, Embase, Pubmed, CNKI, and all relevant studies had been identified by the Cochrane Library. The following key words were used: “comparative studies”, “retropubic”, “open”, “radical prostatectomy “, “Da Vinci”, “robot-assisted”, and “prostate cancer”.

### Data extraction and outcomes of interest

Two of the authors(JKH and TK) extracted data from the selected studies including: author identification, country, publication year, study design, age, No. of patients, operative approaches were mentioned previously, and results of intervention. All disagreements about eligibility were reached a consensus through authors discussion. Perioperative outcomes including operative time, EBL, LOS, overall complications, and oncological outcomes were compared between the two methods from all the studies that were finally selected. Overall complications were graded on the basis of the Clavien-Dindo system [103].

### Inclusion criteria and exclusion criteria

Studies should satisfy the following requirements: (1) to compare RARP with RRP, (2) to display on outcome of two approaches, (3) to document the surgery as RARP or RRP, (4) to clearly document indications for prostatectomy with prostate cancer. Studies will be excluded if (1) the study was not satisfied inclusion criteria or (2) the outcomes of literature were not mentioned or the parameters were impossible to analysis for either RARP or RRP from the published findings and (3) studies focusing on pure robot surgery system and/or on single-site techniques.

### Study quality assessment and level of evidence

In accordance with the criteria of Centre for Evidence-Based Medicine in Oxford, we evaluated the level of evidence(LOE) of included sixteen studies. The Jaded Score was applied to evaluated the methodological quality of RCTs [104]. The Newcastle-Ottawa Scale(NOS) was applied to assessed the methodological quality of non-RCTs observational studies [86, 105]. Two authors(JKH and TK) evaluated the quality of the studies and discrepancies were rechecked by the third reviewer(CZQ) and consensus was achieved by discussion.

### Statistical analysis

All meta-analysis were conducted by Review Manger 5.3(Cochrane Collaboration, Oxford, UK). Continuous and dichotomous variables were calculated by weighted mean differences (WMDs) and odds ratios(ORs). All analysis results were reported with 95% confidence intervals(CIs). I^2^ test and chi-square-based Q test were applied to evaluated the quantity of heterogeneity, and when I^2^ > 50%, the evidence was considered to have substantial heterogeneity, the random- effects(RE) model would be applied, otherwise, the fixed effects(FE) model was applied. The presence of publication bias was evaluated by Egger's test and funnel plot. Sensitivity analysis was used to estimate the influence of studies with a high risk of bias on the overall effect.

## SUPPLEMENTARY MATERIALS TABLES



## References

[1] Siegel RL, Miller KD, Jemal A (2015). Cancer statistics, 2015. CA Cancer J Clin.

[2] Heidenreich A, Bellmunt J, Bolla M, Joniau S, Mason M, Matveev V, Mottet N, Schmid HP, van der Kwast T, Wiegel T, Zattoni F (2011). EAU guidelines on prostate cancer. Part 1: screening, diagnosis, and treatment of clinically localised disease. Eur Urol.

[3] Gratzke C, Dovey Z, Novara G, Geurts N, De Groote R, Schatteman P, de Naeyer G, Gandaglia G, Early Mottrie A (2015). Catheter Removal after Robot-assisted Radical Prostatectomy: Surgical Technique and Outcomes for the Aalst Technique (ECaRemA Study). Eur Urol.

[4] Allan C, Ilic D (2015). Laparoscopic versus Robotic-Assisted Radical Prostatectomy for the Treatment of Localised Prostate Cancer: A Systematic Review. Urol Int.

[5] Tewari A, Sooriakumaran P, Bloch DA, Seshadri-Kreaden U, Hebert AE, Wiklund P (2012). Positive surgical margin and perioperative complication rates of primary surgical treatments for prostate cancer: a systematic review and meta-analysis comparing retropubic, laparoscopic, and robotic prostatectomy. Eur Urol.

[6] Novara G, Ficarra V, Mocellin S, Ahlering TE, Carroll PR, Graefen M, Guazzoni G, Menon M, Patel VR, Shariat SF, Tewari AK, Van Poppel H, Zattoni F (2012). Systematic review and meta-analysis of studies reporting oncologic outcome after robot-assisted radical prostatectomy. Eur Urol.

[7] Novara G, Ficarra V, Rosen RC, Artibani W, Costello A, Eastham JA, Graefen M, Guazzoni G, Shariat SF, Stolzenburg JU, Van Poppel H, Zattoni F, Montorsi F (2012). Systematic review and meta-analysis of perioperative outcomes and complications after robot-assisted radical prostatectomy. Eur Urol.

[8] Ball AJ GB, Fabrizio MD, Davis JW, Given RW, Lynch DF, Shaves M, Schellhammer PF (2006). Prospective longitudinal comparative study of early health-related quality-of-life outcomes in patients undergoing surgical treatment for localized prostate cancer: a short-term evaluation of five approaches from a single institution. J Endourol.

[9] Ahlering TE, Woo D, Eichel L, Lee DI, Edwards R, Skarecky DW (2004). Robot-assisted versus open radical prostatectomy: a comparison of one surgeon’s outcomes. Urology.

[10] Alemozaffar M, Sanda M, Yecies D, Mucci LA, Stampfer MJ, Kenfield SA (2015). Benchmarks for operative outcomes of robotic and open radical prostatectomy: results from the Health Professionals Follow-up Study. Eur Urol.

[11] Bae JJ, Choi SH, Kwon TG, Kim TH (2012). Advantages of robot-assisted laparoscopic radical prostatectomy in obese patients: comparison with the open procedure. Korean J Urol.

[12] Barocas DA, Salem S, Kordan Y, Herrell SD, Chang SS, Clark PE, Davis R, Baumgartner R, Phillips S, Cookson MS, Smith JA Jr. (2010). Robotic assisted laparoscopic prostatectomy versus radical retropubic prostatectomy for clinically localized prostate cancer: comparison of short-term biochemical recurrence-free survival. J Urol.

[13] Bolenz C, Gupta A, Hotze T, Ho R, Cadeddu JA, Roehrborn CG, Lotan Y (2010). Cost comparison of robotic, laparoscopic, and open radical prostatectomy for prostate cancer. Eur Urol.

[14] Breyer BN, Davis CB, Cowan JE, Kane CJ, Carroll PR (2010). Incidence of bladder neck contracture after robot-assisted laparoscopic and open radical prostatectomy. BJU Int.

[15] Busch J, Gonzalgo ML, Leva N, Ferrari M, Cash H, Kempkensteffen C, Hinz S, Miller K, Magheli A (2015). Matched comparison of robot-assisted, laparoscopic and open radical prostatectomy regarding pathologic and oncologic outcomes in obese patients. World J Urol.

[16] Busch J, Magheli A, Leva N, Hinz S, Ferrari M, Friedersdorff F, Fuller TF, Miller K, Gonzalgo ML (2014). Matched comparison of outcomes following open and minimally invasive radical prostatectomy for high-risk patients. World J Urol.

[17] Carlsson S, Nilsson AE, Schumacher MC, Jonsson MN, Volz DS, Steineck G, Wiklund PN (2010). Surgery-related complications in 1253 robot-assisted and 485 open retropubic radical prostatectomies at the Karolinska University Hospital, Sweden. Urology.

[18] Chan RC, Barocas DA, Chang SS, Herrell SD, Clark PE, Baumgartner R, Smith JA, Cookson MS (2008). Effect of a large prostate gland on open and robotically assisted laparoscopic radical prostatectomy. BJU Int.

[19] Chino J, Schroeck FR, Sun L, Lee WR, Albala DM, Moul JW, Koontz BF (2009). Robot-assisted laparoscopic prostatectomy is not associated with early postoperative radiation therapy. BJU Int.

[20] Choi D, Kim D, Kyung YS, Lim JH, Song SH, You D, Jeong IG, Kim CS (2012). Clinical experience with limited lymph node dissection for prostate cancer in Korea: single center comparison of 247 open and 354 robot-assisted laparoscopic radical prostatectomy series. Korean J Urol.

[21] Choo MS, Choi WS, Cho SY, Ku JH, Kim HH, Kwak C (2013). Impact of prostate volume on oncological and functional outcomes after radical prostatectomy: robot-assisted laparoscopic versus open retropubic. Korean J Urol.

[22] Chung SD, Kelle JJ, Huang CY, Chen YH, Lin HC (2012). Comparison of 90-day re-admission rates between open retropubic radical prostatectomy (RRP), laparoscopic RP (LRP) and robot-assisted laparoscopic prostatectomy (RALP). BJU Int.

[23] D’Alonzo RC, Gan TJ, Moul JW, Albala DM, Polascik TJ, Robertson CN, Sun L, Dahm P, Habib AS (2009). A retrospective comparison of anesthetic management of robot-assisted laparoscopic radical prostatectomy versus radical retropubic prostatectomy. J Clin Anesth.

[24] Davis JW, Kreaden US, Gabbert J, Thomas R (2014). Learning curve assessment of robot-assisted radical prostatectomy compared with open-surgery controls from the premier perspective database. J Endourol.

[25] Davison BJ, Matthew A, Gardner AM (2014). Prospective comparison of the impact of robotic-assisted laparoscopic radical prostatectomy versus open radical prostatectomy on health-related quality of life and decision regret. Can Urol Assoc J.

[26] Di Pierro GB, Baumeister P, Stucki P, Beatrice J, Danuser H, Mattei A (2011). A prospective trial comparing consecutive series of open retropubic and robot-assisted laparoscopic radical prostatectomy in a centre with a limited caseload. Eur Urol.

[27] Doumerc N, Yuen C, Savdie R, Rahman MB, Rasiah KK, Pe Benito R, Delprado W, Matthews J, Haynes AM, Stricker PD (2010). Should experienced open prostatic surgeons convert to robotic surgery? The real learning curve for one surgeon over 3 years. BJU Int.

[28] Drouin SJ, Comperat E, Varinot J, Vaessen C, Bitker MO, Chartier-Kastler E, Mozer P, Shariat SF, Cussenot O, Roupret M (2014). The surgical approach can be determined from the pathological specimen obtained after open or robot-assisted laparoscopic radical prostatectomy. World J Urol.

[29] Drouin SJ, Vaessen C, Hupertan V, Comperat E, Misrai V, Haertig A, Bitker MO, Chartier-Kastler E, Richard F, Roupret M (2009). Comparison of mid-term carcinologic control obtained after open, laparoscopic, and robot-assisted radical prostatectomy for localized prostate cancer. World J Urol.

[30] Farnham SB, Webster TM, Herrell SD, Smith JA Jr. (2006). Intraoperative blood loss and transfusion requirements for robotic-assisted radical prostatectomy versus radical retropubic prostatectomy. Urology.

[31] Ficarra V, Novara G, Artibani W, Cestari A, Galfano A, Graefen M, Guazzoni G, Guillonneau B, Menon M, Montorsi F, Patel V, Rassweiler J, Van Poppel H (2009). Retropubic, laparoscopic, and robot-assisted radical prostatectomy: a systematic review and cumulative analysis of comparative studies. Eur Urol.

[32] Fracalanza S, Ficarra V, Cavalleri S, Galfano A, Novara G, Mangano A, Plebani M, Artibani W (2008). Is robotically assisted laparoscopic radical prostatectomy less invasive than retropubic radical prostatectomy? Results from a prospective, unrandomized, comparative study. BJU Int.

[33] Froehner M, Koch R, Leike S, Novotny V, Twelker L, Wirth MP (2013). Urinary tract-related quality of life after radical prostatectomy: open retropubic versus robot-assisted laparoscopic approach. Urol Int.

[34] Gagnon LO, Goldenberg SL, Lynch K, Hurtado A, Gleave ME (2014). Comparison of open and robotic-assisted prostatectomy: The University of British Columbia experience. Can Urol Assoc J.

[35] Gandaglia G, Sammon JD, Chang SL, Choueiri TK, Hu JC, Karakiewicz PI, Kibel AS, Kim SP, Konijeti R, Montorsi F, Nguyen PL, Sukumar S, Menon M (2014). Comparative effectiveness of robot-assisted and open radical prostatectomy in the postdissemination era. J Clin Oncol.

[36] Geraerts I, Van Poppel H, Devoogdt N, Van Cleynenbreugel B, Joniau S, Van Kampen M (2013). Prospective evaluation of urinary incontinence, voiding symptoms and quality of life after open and robot-assisted radical prostatectomy. BJU Int.

[37] Haglind E, Carlsson S, Stranne J, Wallerstedt A, Wilderang U, Thorsteinsdottir T, Lagerkvist M, Damber JE, Bjartell A, Hugosson J, Wiklund P, Steineck G, committee Ls (2015). Urinary Incontinence and Erectile Dysfunction After Robotic Versus Open Radical Prostatectomy: A Prospective, Controlled, Nonrandomised Trial. Eur Urol.

[38] Hall RM, Linklater N, Coughlin G (2014). Robotic and open radical prostatectomy in the public health sector: cost comparison. ANZ J Surg.

[39] Harty NJ, Kozinn SI, Canes D, Sorcini A, Moinzadeh A (2013). Comparison of positive surgical margin rates in high risk prostate cancer: open versus minimally invasive radical prostatectomy. Int Braz J Urol.

[40] Hong JY, Kim JY, Choi YD, Rha KH, Yoon SJ, Kil HK (2010). Incidence of venous gas embolism during robotic-assisted laparoscopic radical prostatectomy is lower than that during radical retropubic prostatectomy. Br J Anaesth.

[41] Hu JC, Gandaglia G, Karakiewicz PI, Nguyen PL, Trinh QD, Shih YC, Abdollah F, Chamie K, Wright JL, Ganz PA, Sun M (2014). Comparative effectiveness of robot-assisted versus open radical prostatectomy cancer control. Eur Urol.

[42] Kim SC, Song C, Kim W, Kang T, Park J, Jeong IG, Lee S, Cho YM, Ahn H (2011). Factors determining functional outcomes after radical prostatectomy: robot-assisted versus retropubic. Eur Urol.

[43] Koo KC, Tuliao P, Yoon YE, Chung BH, Hong SJ, Yang SC, Rha KH (2014). Robot-assisted radical prostatectomy in the Korean population: a 5-year propensity-score matched comparative analysis versus open radical prostatectomy. Int J Urol.

[44] Kordan Y, Barocas DA, Altamar HO, Clark PE, Chang SS, Davis R, Herrell SD, Baumgartner R, Mishra V, Chan RC, Smith JA Jr, Cookson MS (2010). Comparison of transfusion requirements between open and robotic-assisted laparoscopic radical prostatectomy. BJU Int.

[45] Korets R, Weinberg AC, Alberts BD, Woldu SL, Mann MJ, Badani KK (2014). Utilization and timing of blood transfusions following open and robot-assisted radical prostatectomy. J Endourol.

[46] Krambeck AE, DiMarco DS, Rangel LJ, Bergstralh EJ, Myers RP, Blute ML, Gettman MT (2009). Radical prostatectomy for prostatic adenocarcinoma: a matched comparison of open retropubic and robot-assisted techniques. BJU Int.

[47] Laurila TA, Huang W, Jarrard DF (2009). Robotic-assisted laparoscopic and radical retropubic prostatectomy generate similar positive margin rates in low and intermediate risk patients. Urol Oncol.

[48] Ludovico GM, Dachille G, Pagliarulo G, D’Elia C, Mondaini N, Gacci M, Detti B, Malossini G, Bartoletti R, Cai T (2013). Bilateral nerve sparing robotic-assisted radical prostatectomy is associated with faster continence recovery but not with erectile function recovery compared with retropubic open prostatectomy: the need for accurate selection of patients. Oncol Rep.

[49] Magheli A, Gonzalgo ML, Su LM, Guzzo TJ, Netto G, Humphreys EB, Han M, Partin AW, Pavlovich CP (2011). Impact of surgical technique (open vs laparoscopic vs robotic-assisted) on pathological and biochemical outcomes following radical prostatectomy: an analysis using propensity score matching. BJU Int.

[50] Malcolm JB, Fabrizio MD, Barone BB, Given RW, Lance RS, Lynch DF, Davis JW, Shaves ME, Schellhammer PF (2010). Quality of life after open or robotic prostatectomy, cryoablation or brachytherapy for localized prostate cancer. J Urol.

[51] Miller J, Smith A, Kouba E, Wallen E, Pruthi RS (2007). Prospective evaluation of short-term impact and recovery of health related quality of life in men undergoing robotic assisted laparoscopic radical prostatectomy versus open radical prostatectomy. J Urol.

[52] Musch M, Roggenbuck U, Klevecka V, Loewen H, Janowski M, Davoudi Y, Kroepfl D (2013). Does changeover by an experienced open prostatic surgeon from open retropubic to robot-assisted laparoscopic prostatectomy mean a step forward or backward?. ISRN Oncol.

[53] Nelson B, Kaufman M, Broughton G, Cookson MS, Chang SS, Herrell SD, Baumgartner RG, Smith JA Jr (2007). Comparison of length of hospital stay between radical retropubic prostatectomy and robotic assisted laparoscopic prostatectomy. J Urol.

[54] Niklas C, Saar M, Berg B, Steiner K, Janssen M, Siemer S, Stockle M, Ohlmann CH (2015). da Vinci and Open Radical Prostatectomy: Comparison of Clinical Outcomes and Analysis of Insurance Costs. Urol Int.

[55] O’Neil B, Koyama T, Alvarez J, Conwill RM, Albertsen PC, Cooperberg MR, Goodman M, Greenfield S, Hamilton AS, Hoffman KE, Hoffman RM, Kaplan SH, Stanford JL (2015). The Comparative Harms of Open and Robotic Prostatectomy in Population Based Samples. J Urol.

[56] Ong WL, Evans SM, Spelman T, Kearns PA, Murphy DG, Millar JL (2015). Comparison of oncological and health related quality of life (HRQOL) outcomes between open (ORP) and robotic-assisted radical prostatectomy (RARP) for localized prostate cancer - findings from the population-based Victorian Prostate Cancer Registry (PCR). BJU Int.

[57] Park J, Yoo DS, Song C, Park S, Park S, Kim SC, Cho Y, Ahn H (2014). Comparison of oncological outcomes between retropubic radical prostatectomy and robot-assisted radical prostatectomy: an analysis stratified by surgical experience. World J Urol.

[58] Pierorazio PM, Mullins JK, Eifler JB, Voth K, Hyams ES, Han M, Pavlovich CP, Bivalacqua TJ, Partin AW, Allaf ME, Schaeffer EM (2013). Contemporaneous comparison of open vs minimally-invasive radical prostatectomy for high-risk prostate cancer. BJU Int.

[59] Pilecki MA, McGuire BB, Jain U, Kim JY, Nadler RB (2014). National multi-institutional comparison of 30-day postoperative complication and readmission rates between open retropubic radical prostatectomy and robot-assisted laparoscopic prostatectomy using NSQIP. J Endourol.

[60] Porcaro AB, Molinari A, Terrin A, De Luyk N, Baldassarre R, Brunelli M, Cavalleri S, Cerruto MA, Gelati M, Salvagno GL, Guidi GC, Artibani W (2015). Robotic-assisted radical prostatectomy is less stressful than the open approach: results of a contemporary prospective study evaluating pathophysiology of cortisol stress-related kinetics in prostate cancer surgery. J Robot Surg.

[61] Ritch CR, You C, May AT, Herrell SD, Clark PE, Penson DF, Chang SS, Cookson MS, Smith JA Jr, Barocas DA (2014). Biochemical recurrence-free survival after robotic-assisted laparoscopic vs open radical prostatectomy for intermediate- and high-risk prostate cancer. Urology.

[62] Rocco B, Matei DV, Melegari S, Ospina JC, Mazzoleni F, Errico G, Mastropasqua M, Santoro L, Detti S, de Cobelli O (2009). Robotic vs open prostatectomy in a laparoscopically naive centre: a matched-pair analysis. BJU Int.

[63] Rush S, Alibhai SM, Xu L, Xu W, Louis AS, Matthew AG, Nesbitt M, Finelli A, Fleshner NE, Hamilton RJ, Kulkarni G, Zlotta A, Jewett MA (2015). Health-related quality of life in robotic versus open radical prostatectomy. Can Urol Assoc J.

[64] Ryu J, Kwon T, Kyung YS, Hong S, You D, Jeong IG, Kim CS (2013). Retropubic versus robot-assisted laparoscopic prostatectomy for prostate cancer: a comparative study of postoperative complications. Korean J Urol.

[65] Schroeck FR, Sun L, Freedland SJ, Albala DM, Mouraviev V, Polascik TJ, Moul JW (2008). Comparison of prostate-specific antigen recurrence-free survival in a contemporary cohort of patients undergoing either radical retropubic or robot-assisted laparoscopic radical prostatectomy. BJU Int.

[66] Shapiro EY, Scarberry K, Patel T, Bergman A, Ahn JJ, Sahi N, RoyChoudhury A, Deutch I, McKiernan JM, Benson MC, Badani KK (2014). Comparison of robot-assisted and open retropubic radical prostatectomy for risk of biochemical progression in men with positive surgical margins. J Endourol.

[67] Silberstein JL, Su D, Glickman L, Kent M, Keren-Paz G, Vickers AJ, Coleman JA, Eastham JA, Scardino PT, Laudone VP (2013). A case-mix-adjusted comparison of early oncological outcomes of open and robotic prostatectomy performed by experienced high volume surgeons. BJU Int.

[68] Silberstein JL, Vickers AJ, Power NE, Parra RO, Coleman JA, Pinochet R, Touijer KA, Scardino PT, Eastham JA, Laudone VP (2012). Pelvic lymph node dissection for patients with elevated risk of lymph node invasion during radical prostatectomy: comparison of open, laparoscopic and robot-assisted procedures. J Endourol.

[69] Smith JA Jr, Chan RC, Chang SS, Herrell SD, Clark PE, Baumgartner R, Cookson MS (2007). A comparison of the incidence and location of positive surgical margins in robotic assisted laparoscopic radical prostatectomy and open retropubic radical prostatectomy. J Urol.

[70] Son SJ, Lee SC, Jeong CW, Jeong SJ, Byun SS, Lee SE (2013). Comparison of continence recovery between robot-assisted laparoscopic prostatectomy and open radical retropubic prostatectomy: a single surgeon experience. Korean J Urol.

[71] Stranne J, Johansson E, Nilsson A, Bill-Axelson A, Carlsson S, Holmberg L, Johansson JE, Nyberg T, Ruutu M, Wiklund NP, Steineck G (2010). Inguinal hernia after radical prostatectomy for prostate cancer: results from a randomized setting and a nonrandomized setting. Eur Urol.

[72] Sugihara T, Yasunaga H, Horiguchi H, Matsui H, Fujimura T, Nishimatsu H, Fukuhara H, Kume H, Changhong Y, Kattan MW, Fushimi K, Homma Y (2014). Robot-assisted versus other types of radical prostatectomy: population-based safety and cost comparison in Japan, 2012-2013. Cancer Sci.

[73] Tewari A, Srivasatava A, Menon M (2003). A prospective comparison of radical retropubic and robot-assisted prostatectomy: experience in one institution. BJU International.

[74] Vora AA, Marchalik D, Kowalczyk KJ, Nissim H, Bandi G, McGeagh KG, Lynch JH, Ghasemian SR, Verghese M, Venkatesan K, Borges P, Uchio EM, Hwang JJ (2013). Robotic-assisted prostatectomy and open radical retropubic prostatectomy for locally-advanced prostate cancer: multi-institution comparison of oncologic outcomes. Prostate Int.

[75] Wallerstedt A, Tyritzis SI, Thorsteinsdottir T, Carlsson S, Stranne J, Gustafsson O, Hugosson J, Bjartell A, Wilderang U, Wiklund NP, Steineck G, Haglind E, committee Ls (2015). Short-term results after robot-assisted laparoscopic radical prostatectomy compared to open radical prostatectomy. Eur Urol.

[76] White MA, De Haan AP, Stephens DD, Maatman TK, Maatman TJ (2009). Comparative analysis of surgical margins between radical retropubic prostatectomy and RALP: are patients sacrificed during initiation of robotics program?. Urology.

[77] Williams SB, Chen MH, D’Amico AV, Weinberg AC, Kacker R, Hirsch MS, Richie JP, Hu JC (2010). Radical retropubic prostatectomy and robotic-assisted laparoscopic prostatectomy: likelihood of positive surgical margin(s). Urology.

[78] Wood DP, Schulte R, Dunn RL, Hollenbeck BK, Saur R, Wolf JS Jr, Montie JE (2007). Short-term health outcome differences between robotic and conventional radical prostatectomy. Urology.

[79] Yi JS, Kwak C, Kim HH, Ku JH (2010). Surgical clip-related complications after radical prostatectomy. Korean J Urol.

[80] Akand M CO, Avci E, Duman I, Erdogru T (2015). Open, laparoscopic and robot-assisted laparoscopic radical prostatectomy: comparative analysis of operative and pathologic outcomes for three techniques with a single surgeon’s experience. Eur Rev Med Pharmacol Sci.

[81] Lo KL NC, Lam CN, Hou SS, To KF, Yip SK Short-term outcome of patients with robot-assisted versus open radical prostatectomy: for localised carcinoma of prostate. Hong Kong Med J.

[82] Menon M TA, Baize B, Guillonneau B, Vallancien G (2002). Prospective comparison of radical retropubic prostatectomy and robot-assisted anatomic prostatectomy: the Vattikuti Urology Institute experience. Urology.

[83] Minniti D, Chiado Piat S, Di Novi C (2011). Robot-assisted versus open radical prostatectomy: an evidence-based comparison. Technol Health Care.

[84] Ou YC, Yang CR, Wang J, Cheng CL, Patel VR (2009). Comparison of robotic-assisted versus retropubic radical prostatectomy performed by a single surgeon. Anticancer Res.

[85] Truesdale MD, Lee DJ, Cheetham PJ, Hruby GW, Turk AT, Badani KK (2010). Assessment of lymph node yield after pelvic lymph node dissection in men with prostate cancer: a comparison between robot-assisted radical prostatectomy and open radical prostatectomy in the modern era. J Endourol.

[86] Feng MX, Hong JX, Wang Q, Fan YY, Yuan CT, Lei XH, Zhu M, Qin A, Chen HX, Hong D (2016). Dihydroartemisinin prevents breast cancer-induced osteolysis via inhibiting both breast caner cells and osteoclasts. Sci Rep.

[87] Clark HD, Wells GA, Huët C, McAlister FA, Salmi LR, Fergusson D, Laupacis A (1999). Assessing the Quality of Randomized Trials: Reliability of the Jadad Scale.

[88] Vasdev N, Bishop C, Kass-Iliyya A, Hamid S, McNicholas TA, Prasad V, Mohan SG, Lane T, Boustead G, Adshead JM (2013). Developing a robotic prostatectomy service and a robotic fellowship programme - defining the learning curve. Curr Urol.

[89] Heidenreich A PD (2015). Extended pelvic lymphadenectomy in prostate cancer: Practice makes perfect. Can Urol Assoc J.

[90] Donat SM (2007). Standards for surgical complication reporting in urologic oncology: time for a change. Urology.

[91] Ficarra V, Novara G, Rosen RC, Artibani W, Carroll PR, Costello A, Menon M, Montorsi F, Patel VR, Stolzenburg JU, Van der Poel H, Wilson TG, Zattoni F (2012). Systematic review and meta-analysis of studies reporting urinary continence recovery after robot-assisted radical prostatectomy. Eur Urol.

[92] Patel VR, Samavedi S, Bates AS, Kumar A, Coelho R, Rocco B, Palmer K (2015). Dehydrated Human Amnion/Chorion Membrane Allograft Nerve Wrap Around the Prostatic Neurovascular Bundle Accelerates Early Return to Continence and Potency Following Robot-assisted Radical Prostatectomy: Propensity Score-matched Analysis. Eur Urol.

[93] Tan G, Srivastave A, Grover S, Peters D, Dorsey P, Scott A, Jhaveri J, Tilki D, Te A, Tewari A (2010). Optimizing Vesicourethral Anastomosis Healing After Robot-Assisted Laparoscopic Radical Prostatectomy: Lessons Learned from Three Techniques in 1900 Patients. J Endourol.

[94] Wiltz AL, Shikanov S, Eggener SE, Katz MH, Thong AE, Steinberg GD, Shalhav AL, Zagaja GP, Zorn KC (2009). Robotic radical prostatectomy in overweight and obese patients: oncological and validated-functional outcomes. Urology.

[95] Froehner M KA, Koch R, Baretton GB, Hakenberg OW, Wirth MP (2014). A combined index to classify prognostic comorbidity in candidates for radical prostatectomy. BMC Urol.

[96] Skolarus TA, Hedgepeth RC, Zhang Y, Weizer AZ, Montgomery JS, Miller DC, Wood DP Jr, Hollenbeck BK (2010). Does robotic technology mitigate the challenges of large prostate size?. Urology.

[97] Yasui T TK, Kurokawa S, Okada A, Mizuno K, Umemoto Y, Kawai N, Sasaki S, Hayashi Y, Kojima Y, Kohri K (2014). Impact of prostate weight on perioperative outcomes of robot-assisted laparoscopic prostatectomy with a posterior approach to the seminal vesicle. BMC Urol.

[98] Ficarra V, Novara G, Ahlering TE, Costello A, Eastham JA, Graefen M, Guazzoni G, Menon M, Mottrie A, Patel VR, Van der Poel H, Rosen RC, Tewari AK (2012). Systematic review and meta-analysis of studies reporting potency rates after robot-assisted radical prostatectomy. Eur Urol.

[99] Jeong SJ, Yi J, Chung MS, Kim DS, Lee WK, Park H, Yoon CY, Hong SK, Byun SS, Lee SE (2011). Early recovery of urinary continence after radical prostatectomy: correlation with vesico-urethral anastomosis location in the pelvic cavity measured by postoperative cystography. Int J Urol.

[100] Moran PS, O’Neill M, Teljeur C, Flattery M, Murphy LA, Smyth G, Ryan M (2013). Robot-assisted radical prostatectomy compared with open and laparoscopic approaches: a systematic review and meta-analysis. Int J Urol.

[101] Robertson C, Close A, Fraser C, Gurung T, Jia X, Sharma P, Vale L, Ramsay C, Pickard R (2013). Relative effectiveness of robot-assisted and standard laparoscopic prostatectomy as alternatives to open radical prostatectomy for treatment of localised prostate cancer: a systematic review and mixed treatment comparison meta-analysis. BJU Int.

[102] Liberati A, Altman DG, Tetzlaff J, Mulrow C, Gotzsche PC, Ioannidis JP, Clarke M, Devereaux PJ, Kleijnen J, Moher D (2009). The PRISMA statement for reporting systematic reviews and meta-analyses of studies that evaluate health care interventions: explanation and elaboration. J Clin Epidemiol.

[103] Dindo D, Demartines N, Clavien PA (2004). Classification of Surgical Complications. Annals of Surgery.

[104] Clark HD, Wells GA, Huet C, McAlister FA, Salmi LR, Fergusson D, Laupacis A (1999). Assessing the quality of randomized trials: reliability of the Jadad scale. Control Clin Trials.

[105] Taggart DP, D’Amico R, Altman DG (2001). Effect of arterial revascularisation on survival: a systematic review of studies comparing bilateral and single internal mammary arteries. The Lancet.

