# Supplementary material for: Robotic vs. Retropubic radical prostatectomy in prostate cancer: A systematic review and a meta-analysis update
Source: Oncotarget. 2016 Nov 12;8(19):32237–57. doi: 10.18632/oncotarget.13332 (PMC5458281; doi:10.18632/oncotarget.13332)
Supplement: Supplementary file 1 [file oncotarget-08-32237-s001.pdf]

Robotic *vs.* Retropubic radical prostatectomy in prostate cancer: A systematic review and a meta-analysis update

Supplementary Material

Study or Subgroup	RARP Mean	RARP SD	RARP Total	RRP Mean	RRP SD	RRP Total	Weight	Mean Difference IV, Random, 95% CI
Bae, 2012	65.39	6.64	111	66.07	5.04	70	2.8%	-0.68 [-2.39, 1.03]
Barocas,2010	61	7.3	1413	62	7.3	491	3.5%	-1.00 [-1.75, -0.25]
Breyer,2010	59.7	7.1	293	59.2	6.7	695	3.4%	0.50 [-0.45, 1.45]
Busch 2014	64.4	0.6	110	64.2	0.6	110	3.7%	0.20 [0.04, 0.36]
Busch,2015	61.9	0.48	194	61.3	0.47	194	3.7%	0.60 [0.51, 0.69]
Choi,2012	64.03	7.47	354	66.38	5.86	247	3.3%	-2.35 [-3.42, -1.28]
Choo,2013	66	7.75	77	67	6.25	176	2.6%	-1.00 [-2.96, 0.96]
D'Alonzo,2009	59	6.6	219	60	6.9	251	3.2%	-1.00 [-2.22, 0.22]
Davis 2014	61	7.1	27348	63.5	8.2	13480	3.7%	-2.50 [-2.66, -2.34]
Davison 2014	61.3	6.4	78	62.8	6.9	73	2.4%	-1.50 [-3.63, 0.63]
Farnham,2006	59	7	176	60	7.8	103	2.7%	-1.00 [-2.83, 0.83]
Gagnon 2014	64.22	7.07	200	64.65	7.2	200	3.0%	-0.43 [-1.83, 0.97]
Geraerts 2013	61.48	6.08	64	62.22	6.12	116	2.6%	-0.74 [-2.60, 1.12]
Harty 2013	61.3	6.9	152	59.5	6.5	153	2.9%	1.80 [0.30, 3.30]
Hong,2010	66	6.9	26	68.6	4.9	25	1.6%	-2.60 [-5.87, 0.67]
Kim,2011	64.2	7.3	528	66.5	5.7	235	3.4%	-2.30 [-3.26, -1.34]
Koo 2014	65.6	6.5	175	65.9	6.1	175	3.1%	-0.30 [-1.62, 1.02]
Kordan,2010	60.5	7.2	830	61.5	7.5	414	3.4%	-1.00 [-1.87, -0.13]
Ludovico 2013	68.1	2.3	82	66.7	9.9	48	1.9%	1.40 [-1.44, 4.24]
Magheli,2011	58.3	6.3	522	58.8	6.1	522	3.5%	-0.50 [-1.25, 0.25]
Malcolm,2010	59	6	447	59	7	135	3.1%	0.00 [-1.31, 1.31]
Niklas 2015	63.7	7	932	64.9	6.6	499	3.5%	-1.20 [-1.93, -0.47]
Ong 2015	62.1	6.7	885	62.3	6.7	1117	3.6%	-0.20 [-0.79, 0.39]
OU,2009	67.27	6.21	30	70.03	6.1	30	1.7%	-2.76 [-5.87, 0.35]
Park,2014	64.2	7.4	730	66.8	5.7	277	3.4%	-2.60 [-3.46, -1.74]
Pilecki,2014	61.7	7.2	4374	63.1	7.4	1097	3.6%	-1.40 [-1.89, -0.91]
Porcaro 2015	62.48	6.12	108	65.97	5.6	43	2.5%	-3.49 [-5.52, -1.46]
Rush,2015	59.71	7.03	331	61.49	7.07	643	3.4%	-1.78 [-2.71, -0.85]
Ryu,2013	64.9	7	524	67.9	6.7	341	3.4%	-3.00 [-3.93, -2.07]
Shapiro,2014	61.5	7	108	61.6	7	229	2.9%	-0.10 [-1.70, 1.50]
Smith,2007	60.3	7.3	200	61.1	7.35	200	3.0%	-0.80 [-2.24, 0.64]
Son,2013	65.5	6.7	146	65	6.1	112	2.9%	0.50 [-1.07, 2.07]
Truesdale,2010	59.2	7.1	99	61.7	6.8	217	2.8%	-2.50 [-4.17, -0.83]
**Total (95% CI)**			**41866**			**22718**	**100.0%**	**-1.00 [-1.56, -0.44]**

Heterogeneity: Tau² = 2.21; Chi² = 1260.51, df = 32 (P < 0.00001); I² = 97%
Test for overall effect: Z = 3.48 (P = 0.0005)

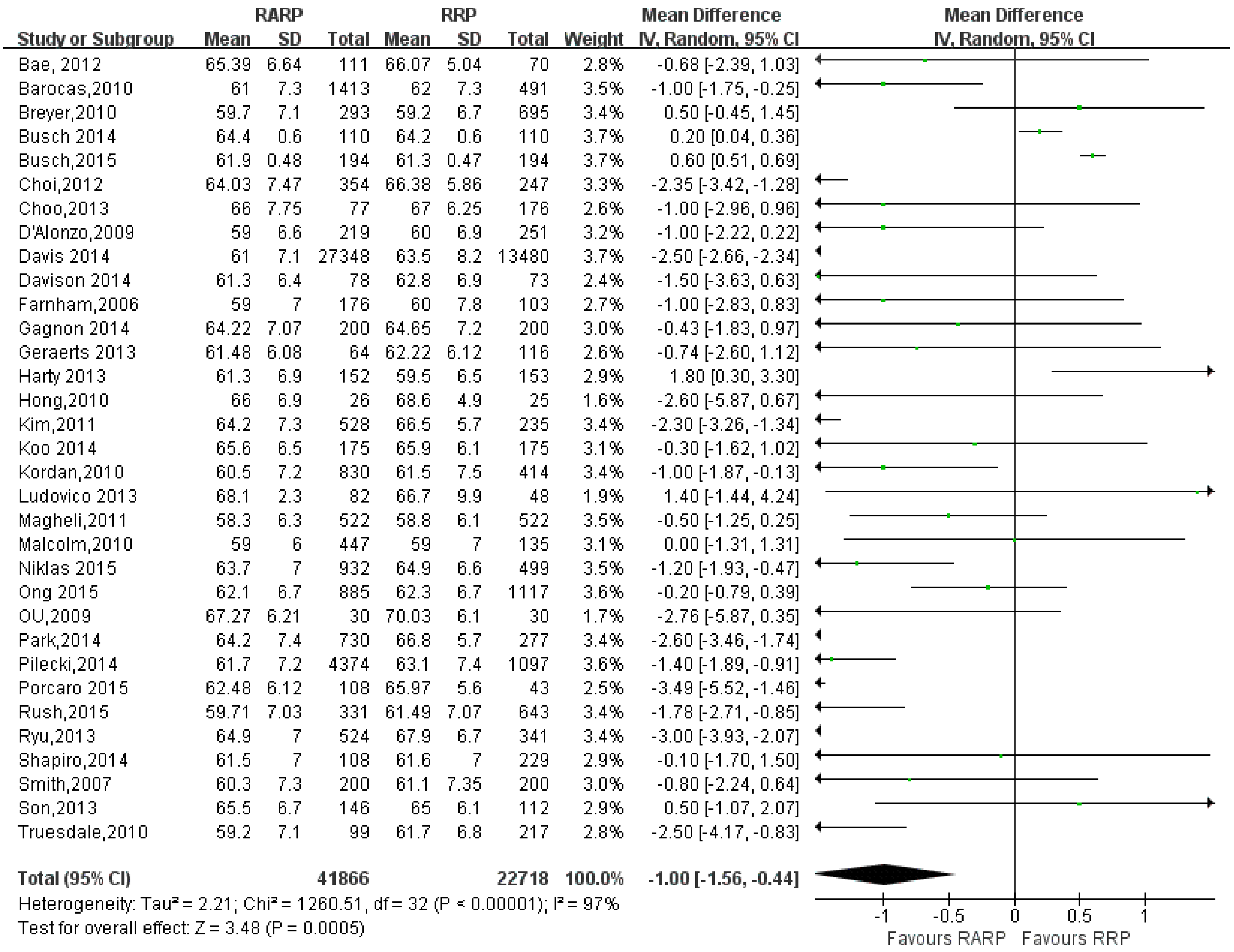

Fig.S1. Forest plot and meta-analysis of age between RARP and RRP. RARP= robot-assisted radical prostatectomy; RRP= retropubic radical prostatectomy.

Study or Subgroup	RARP Mean	RARP SD	RARP Total	RRP Mean	RRP SD	RRP Total	Weight	Mean Difference IV, Random, 95% CI
Bae, 2012	15.96	16.17	111	15.86	17.99	70	1.0%	0.10 [-5.08, 5.28]
Breyer,2010	7.1	5.39	293	7.6	7.26	695	7.0%	-0.50 [-1.32, 0.32]
Busch 2014	11	0.998	110	11.4	0.919	100	8.2%	-0.40 [-0.66, -0.14]
Busch,2015	6.8	0.282	194	6.2	0.245	194	8.4%	0.60 [0.55, 0.65]
Choi,2012	8.41	8.38	354	11.88	13.32	247	4.2%	-3.47 [-5.35, -1.59]
Choo,2013	7.2	13.19	77	7.6	19.33	176	1.4%	-0.40 [-4.50, 3.70]
D'Alonzo,2009	6	3.5	219	7.3	8.1	103	4.7%	-1.30 [-2.93, 0.33]
Farnham,2006	6.5	4.7	176	8.3	8.9	103	4.2%	-1.80 [-3.65, 0.05]
Gagnon 2014	6.57	4.352	200	11.16	11.884	200	4.4%	-4.59 [-6.34, -2.84]
Kim,2011	10.4	16	528	14.6	22.1	235	2.2%	-4.20 [-7.34, -1.06]
Koo 2014	12.3	16.6	175	12.2	16.1	175	1.9%	0.10 [-3.33, 3.53]
Lo,2010	14.2	11.8	20	14.5	14.3	20	0.4%	-0.30 [-8.43, 7.83]
Ludovico 2013	8.3	3.1	82	7.9	2.1	48	6.8%	0.40 [-0.50, 1.30]
Magheli,2011	5.4	3.2	522	5.4	3.2	522	8.0%	0.00 [-0.39, 0.39]
Niklas 2015	8.4	8.9	932	8	8.6	499	6.7%	0.40 [-0.55, 1.35]
OU,2009	16.45	18.8	30	15.89	14.15	30	0.4%	0.56 [-7.86, 8.98]
Park,2014	9.2	9.7	730	10.3	11	277	5.1%	-1.10 [-2.57, 0.37]
Porcaro 2015	6.96	3.44	108	12.64	17.06	43	1.0%	-5.68 [-10.82, -0.54]
Rush,2015	5.89	3.28	331	7.58	5.22	643	7.7%	-1.69 [-2.23, -1.15]
Ryu,2013	10.1	11.4	524	9.7	11.2	341	5.0%	0.40 [-1.14, 1.94]
Smith,2007	6.4	5.3	200	8.3	6.9	200	5.9%	-1.90 [-3.11, -0.69]
Son,2013	13	20.4	146	13	20.4	112	1.0%	0.00 [-5.02, 5.02]
Truesdale,2010	7.04	7.5	99	8.35	7.62	217	4.3%	-1.31 [-3.10, 0.48]
**Total (95% CI)**			**6161**			**5250**	**100.0%**	**-0.93 [-1.47, -0.40]**

Heterogeneity: $Tau^2 = 0.90$; $Chi^2 = 234.69$, df = 22 ($P < 0.00001$); $I^2 = 91\%$
Test for overall effect: Z = 3.40 (P = 0.0007)

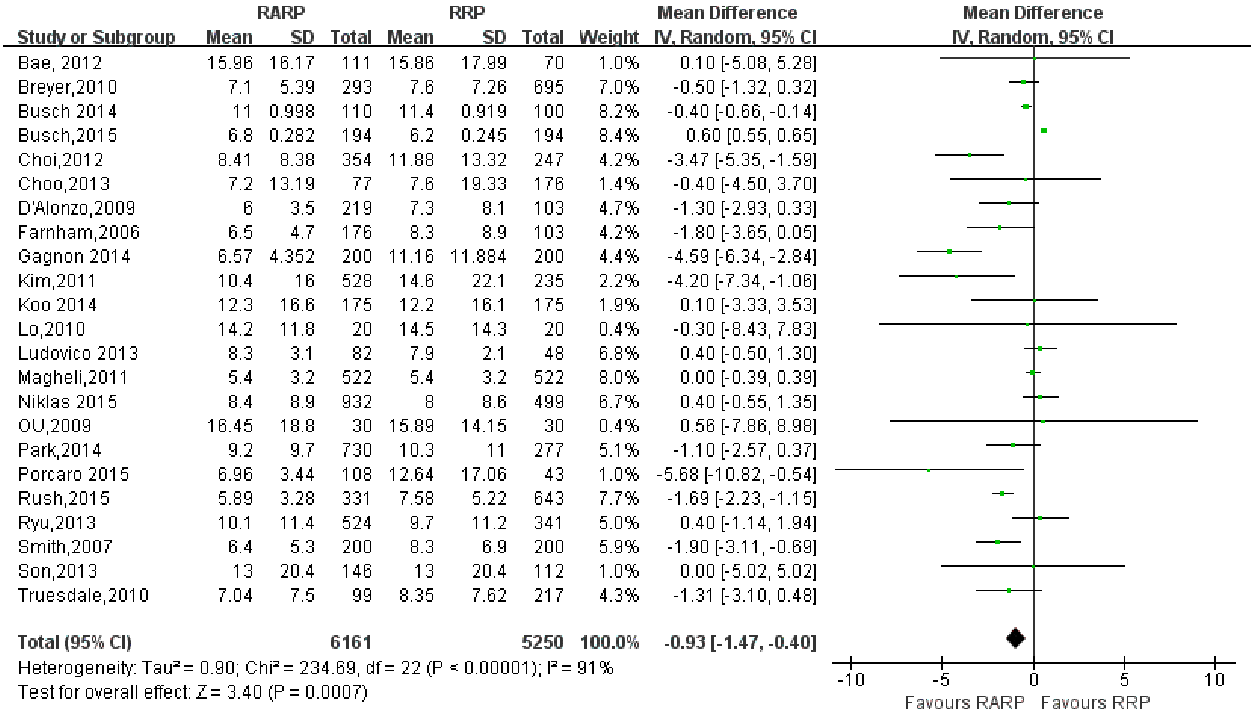

Fig.S2. Forest plot and meta-analysis of the level of pre-PSA between RARP and RRP. RARP= robot-assisted radical prostatectomy; RRP= retropubic radical prostatectomy.

Study or Subgroup	RARP Mean	RARP SD	RARP Total	RRP Mean	RRP SD	RRP Total	Weight	Mean Difference IV, Random, 95% CI
Bae, 2012	23.96	2.3	111	23.13	2.68	70	5.6%	0.83 [0.07, 1.59]
Busch 2014	27.9	0.38	110	27.5	0.44	110	9.0%	0.40 [0.29, 0.51]
Choo,2013	24	2.55	77	24	2.73	176	6.0%	0.00 [-0.70, 0.70]
Fracalanza,2008	25.5	2.7	35	26.4	3.7	26	2.3%	-0.90 [-2.58, 0.78]
Gagnon 2014	27.16	3.27	200	27.23	4.31	200	5.7%	-0.07 [-0.82, 0.68]
Kim,2011	24.5	2.7	528	25.1	3.6	235	7.1%	-0.60 [-1.11, -0.09]
Kordan,2010	28.2	4.2	830	28	4.6	414	7.0%	0.20 [-0.33, 0.73]
Niklas 2015	27.1	3.9	932	27.5	5	499	7.1%	-0.40 [-0.91, 0.11]
OU,2009	24.22	3.16	30	24.09	3.28	30	2.4%	0.13 [-1.50, 1.76]
Park,2014	24.6	2.7	730	25.1	3.4	277	7.5%	-0.50 [-0.95, -0.05]
Pilecki,2014	28.6	4.8	4374	28.5	5.2	1097	8.1%	0.10 [-0.24, 0.44]
Porcaro 2015	25.79	2.78	108	26.81	3.15	43	4.1%	-1.02 [-2.10, 0.06]
Rush 2015	27.3	3.6	331	28.6	4	643	7.2%	-1.30 [-1.80, -0.80]
Ryu,2013	24.6	2.7	524	24.7	2.7	341	7.9%	-0.10 [-0.47, 0.27]
Smith,2007	28.5	4.4	200	27.8	4.8	200	4.9%	0.70 [-0.20, 1.60]
Son,2013	24.5	2.5	146	24.3	2.4	112	6.6%	0.20 [-0.40, 0.80]
Truesdale,2010	24.6	8.3	99	23.1	9.1	217	1.7%	1.50 [-0.53, 3.53]
**Total (95% CI)**			**9365**			**4690**	**100.0%**	**-0.10 [-0.39, 0.20]**

Heterogeneity: $Tau^2 = 0.24$; $Chi^2 = 87.93$, df = 16 ($P < 0.00001$); $I^2 = 82\%$
Test for overall effect: Z = 0.64 (P = 0.52)

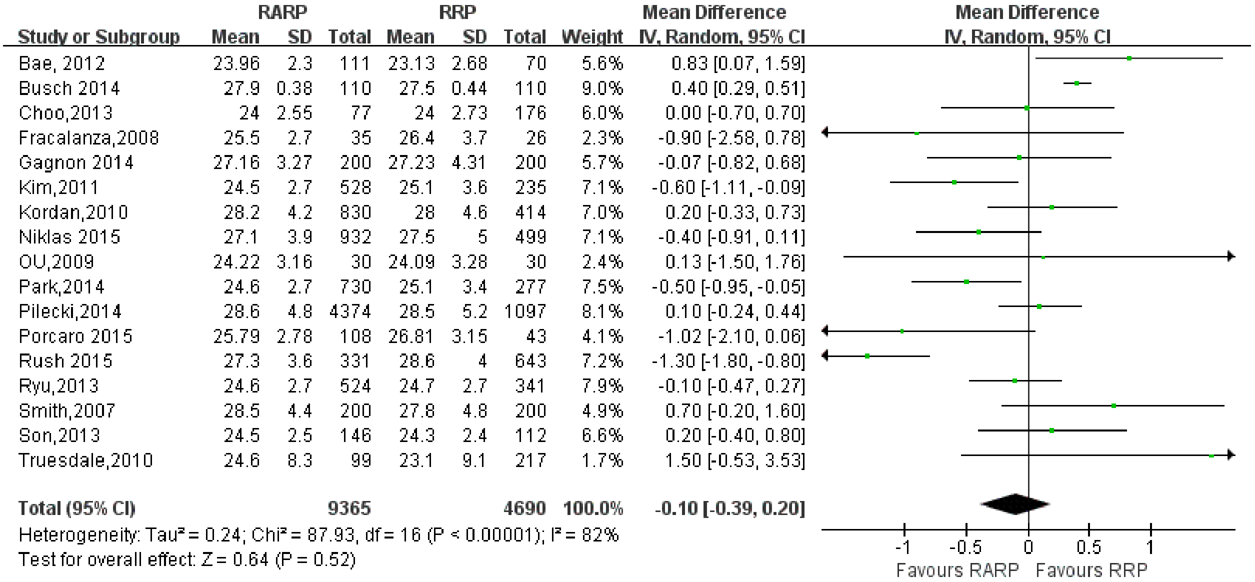

Fig.S3. Forest plot and meta-analysis of BMI between RARP and RRP. RARP= robot-assisted radical prostatectomy; RRP= retropubic radical prostatectomy.

Study or Subgroup	RARP Mean	RARP SD	RARP Total	RRP Mean	RRP SD	RRP Total	Weight	Mean Difference IV, Random, 95% CI
Bae, 2012	33.39	15.53	111	28.83	13.62	70	8.1%	4.56 [0.26, 8.86]
Breyer,2010	34.5	19.34	293	34.3	19.55	695	8.9%	0.20 [-2.45, 2.85]
Choo,2013	41	15.77	77	42	18.82	176	8.0%	-1.00 [-5.49, 3.49]
Gagnon 2014	36.93	23.98	200	32.22	24.09	200	7.9%	4.71 [-0.00, 9.42]
Harty 2013	47.5	14.5	152	39.6	12.2	153	8.7%	7.90 [4.89, 10.91]
Magheli,2011	49	16	522	54	17	522	9.1%	-5.00 [-7.00, -3.00]
Niklas 2015	58.5	31	932	61.8	25	499	8.7%	-3.30 [-6.26, -0.34]
Park,2014	34.5	15.2	730	25.4	16.2	277	9.0%	9.10 [6.90, 11.30]
Porcaro 2015	36.56	16.68	108	38.59	15.89	43	7.3%	-2.03 [-7.73, 3.67]
Ryu,2013	36	16.9	524	36.2	17.8	341	9.0%	-0.20 [-2.58, 2.18]
Smith,2007	53	19.8	200	43.9	22.7	200	8.2%	9.10 [4.93, 13.27]
Son,2013	45.9	16.3	146	41.3	30	112	7.1%	4.60 [-1.55, 10.75]
**Total (95% CI)**			**3995**			**3288**	**100.0%**	**2.35 [-0.92, 5.61]**

Heterogeneity: Tau² = 29.46; Chi² = 136.49, df = 11 (P < 0.00001); I² = 92%
Test for overall effect: Z = 1.41 (P = 0.16)

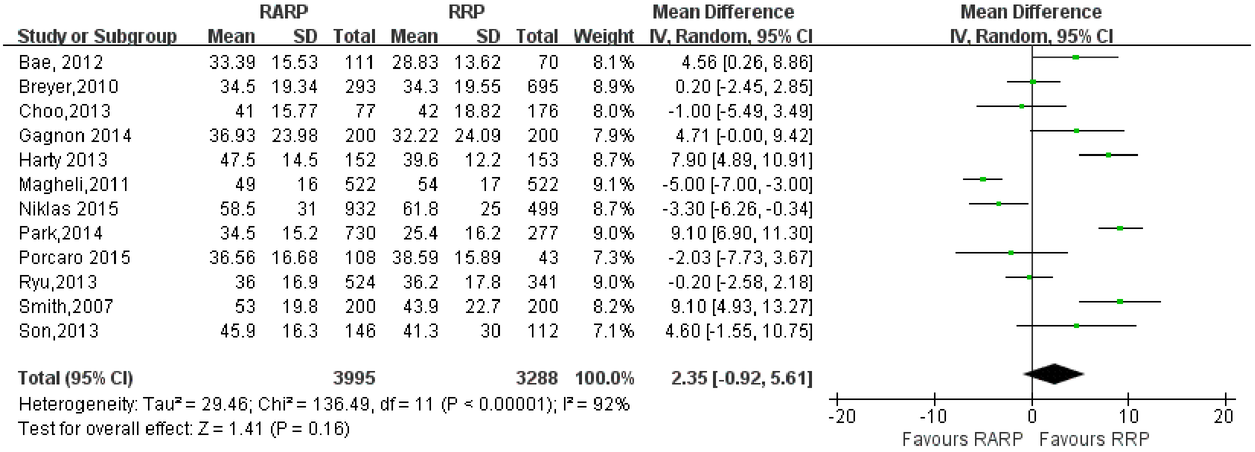

Fig.S4. Forest plot and meta-analysis of prostate volume between RARP and RRP. RARP= robot-assisted radical prostatectomy; RRP= retropubic radical prostatectomy.

Study or Subgroup	RARP Mean	RARP SD	RARP Total	RRP Mean	RRP SD	RRP Total	Weight	Mean Difference IV, Random, 95% CI
Lo,2010	12	7	20	18	7	20	8.3%	-6.00 [-10.34, -1.66]
Nelson,2007	2	0.3	629	4	0.3	374	24.0%	-2.00 [-2.04, -1.96]
Niklas 2015	6.6	6.2	932	12.8	4	499	23.3%	-6.20 [-6.73, -5.67]
OU,2009	7.7	2.08	30	9.2	2.86	30	20.7%	-1.50 [-2.77, -0.23]
Ryu,2013	6.2	2.6	524	7.5	2.4	341	23.7%	-1.30 [-1.64, -0.96]
**Total (95% CI)**			**2135**			**1264**	**100.0%**	**-3.04 [-4.59, -1.49]**

Heterogeneity: Tau² = 2.61; Chi² = 260.52, df = 4 (P < 0.00001); I² = 98%
Test for overall effect: Z = 3.85 (P = 0.0001)

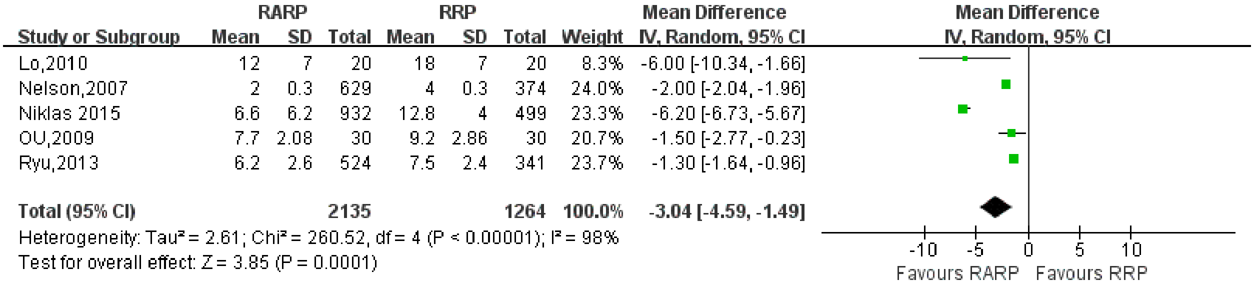

Fig.S5. Forest plot and meta-analysis of the time to remove catheter between RARP and RRP. RARP= robot-assisted radical prostatectomy; RRP= retropubic radical prostatectomy.

Study or Subgroup	RARP Events	RARP Total	RRP Events	RRP Total	Weight	Odds Ratio M-H, Fixed, 95% CI
Ahlering,2004	16	60	9	60	2.7%	2.06 [0.83, 5.12]
Akand 2015	10	79	6	50	2.6%	1.06 [0.36, 3.13]
Choi,2012	229	357	141	247	24.5%	1.34 [0.96, 1.87]
Doumerc,2010	18	212	37	502	8.3%	1.17 [0.65, 2.10]
Drouin 2014	1	73	4	44	2.0%	0.14 [0.02, 1.29]
Drouin,2009	3	71	5	83	1.8%	0.69 [0.16, 2.99]
Fracalanza,2008	4	35	3	26	1.3%	0.99 [0.20, 4.86]
Krambeck,2008	105	294	206	588	36.2%	1.03 [0.77, 1.38]
Ludovico 2013	22	82	11	48	4.2%	1.23 [0.54, 2.83]
Menon,2002	24	30	23	30	1.9%	1.22 [0.36, 4.17]
Tewari,2003	30	200	18	100	8.4%	0.80 [0.42, 1.53]
White,2009	12	50	12	50	3.7%	1.00 [0.40, 2.50]
Williams,2010	596	604	343	346	2.4%	0.65 [0.17, 2.47]
**Total (95% CI)**		**2147**		**2174**	**100.0%**	**1.11 [0.93, 1.31]**
Total events	1070		818			

Heterogeneity: Chi² = 8.84, df = 12 (P = 0.72); I² = 0%
Test for overall effect: Z = 1.14 (P = 0.26)

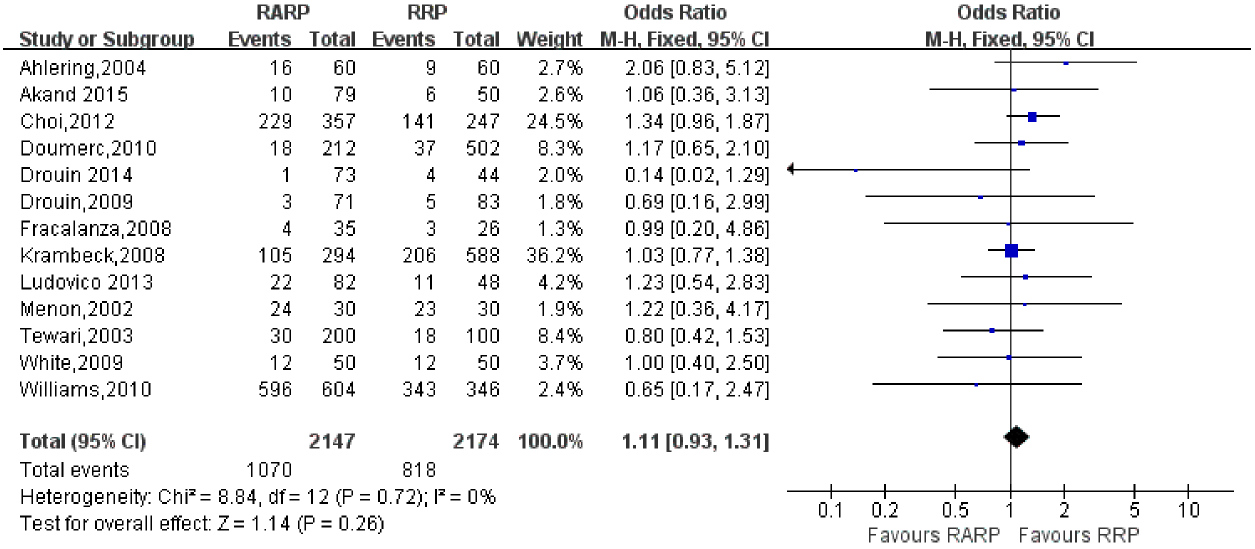

Fig.S6. Forest plot and meta-analysis of pT≤T2a between RARP and RRP. RARP= robot-assisted radical prostatectomy; RRP= retropubic radical prostatectomy.

Study or Subgroup	RARP Events	RARP Total	RRP Events	RRP Total	Weight	Odds Ratio M-H, Fixed, 95% CI
Ahlering,2004	29	60	35	60	7.9%	0.67 [0.32, 1.37]
Akand 2015	15	79	15	50	6.5%	0.55 [0.24, 1.25]
Choi,2012	95	254	65	247	18.1%	1.67 [1.14, 2.45]
Doumerc,2010	12	212	20	502	4.9%	1.45 [0.69, 3.01]
Drouin 2014	3	73	2	44	1.0%	0.90 [0.14, 5.61]
Drouin,2009	10	71	5	83	1.7%	2.56 [0.83, 7.87]
Krambeck,2008	159	294	315	588	42.3%	1.02 [0.77, 1.35]
Ludovico 2013	13	82	8	48	3.7%	0.94 [0.36, 2.47]
Menon,2002	3	30	3	30	1.2%	1.00 [0.19, 5.40]
Tewari,2003	144	200	75	100	12.3%	0.86 [0.50, 1.48]
Williams,2010	3	604	0	346	0.3%	4.03 [0.21, 78.30]
**Total (95% CI)**		**1959**		**2098**	**100.0%**	**1.11 [0.93, 1.33]**
Total events	486		543			

Heterogeneity: Chi² = 13.91, df = 10 (P = 0.18); I² = 28%
Test for overall effect: Z = 1.16 (P = 0.25)

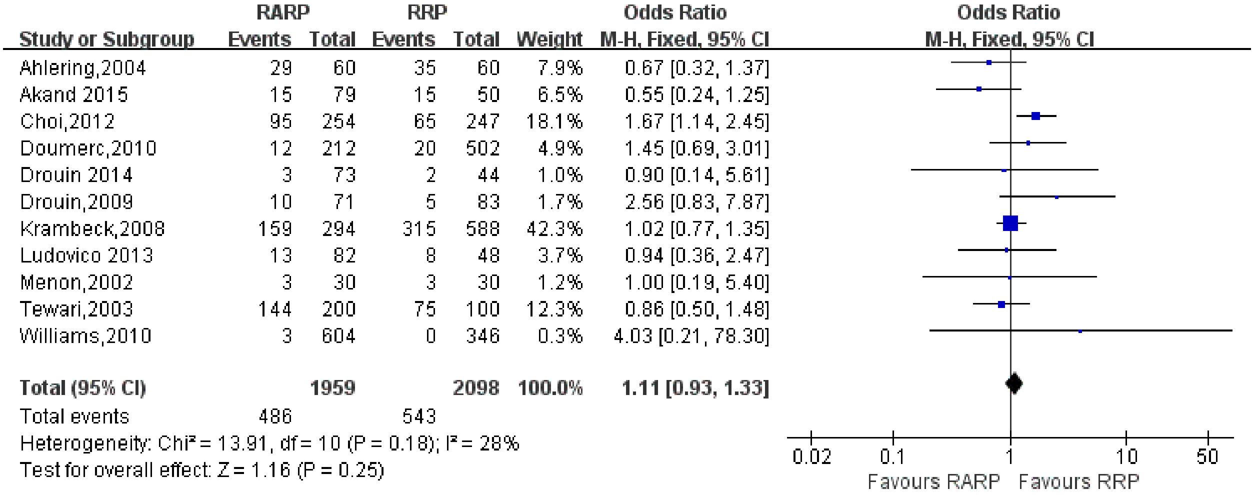

Fig.S7. Forest plot and meta-analysis of pT=T2b between RARP and RRP. RARP= robot-assisted radical prostatectomy; RRP= retropubic radical prostatectomy.

Study or Subgroup	RARP Events	RARP Total	RRP Events	RRP Total	Weight	Odds Ratio M-H, Fixed, 95% CI
Ahlering,2004	15	60	16	60	5.8%	0.92 [0.40, 2.08]
Akand 2015	54	79	29	50	5.4%	1.56 [0.75, 3.26]
Choi,2012	30	254	41	247	17.6%	0.67 [0.41, 1.12]
Doumerc,2010	182	212	445	502	17.9%	0.78 [0.48, 1.25]
Drouin 2014	69	73	38	44	1.2%	2.72 [0.72, 10.25]
Drouin,2009	58	71	73	83	5.9%	0.61 [0.25, 1.49]
Fracalanza,2008	31	35	23	26	1.4%	1.01 [0.21, 4.96]
Krambeck,2008	29	294	67	588	19.3%	0.85 [0.54, 1.35]
Ludovico 2013	49	82	29	48	7.1%	0.97 [0.47, 2.01]
Shapiro,2014	105	108	220	229	1.9%	1.43 [0.38, 5.40]
Son,2013	129	146	102	112	6.4%	0.74 [0.33, 1.69]
Tewari,2003	26	200	7	100	3.9%	1.99 [0.83, 4.75]
White,2009	38	50	38	50	4.4%	1.00 [0.40, 2.50]
Williams,2010	5	604	3	346	1.8%	0.95 [0.23, 4.02]
**Total (95% CI)**		**2268**		**2485**	**100.0%**	**0.93 [0.76, 1.13]**
Total events	820		1131			

Heterogeneity: Chi² = 11.19, df = 13 (P = 0.60); I² = 0%
Test for overall effect: Z = 0.78 (P = 0.44)

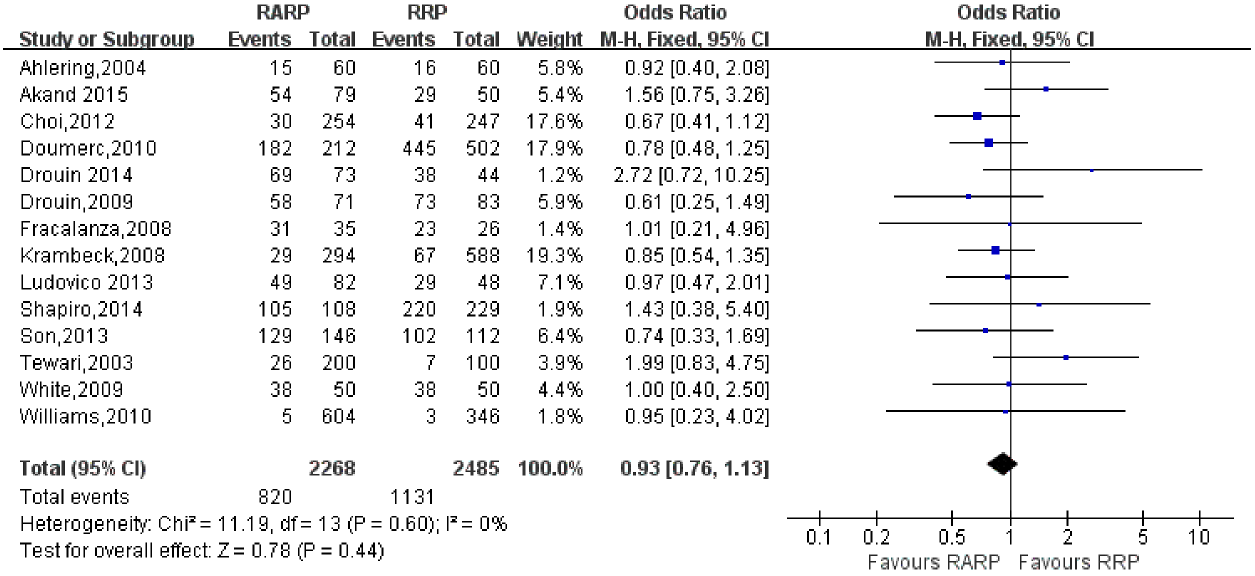

Fig.S8. Forest plot and meta-analysis of pT≥T2c between RARP and RRP. RARP= robot-assisted radical prostatectomy; RRP= retropubic radical prostatectomy.

Study or Subgroup	RARP Mean	RARP SD	RARP Total	RRP Mean	RRP SD	RRP Total	Weight	Mean Difference IV, Fixed, 95% CI
Choi,2012	5.83	3.28	354	4.59	2.95	247	75.6%	1.24 [0.74, 1.74]
Koo 2014	12.5	7.8	175	3.8	5.9	175	9.1%	8.70 [7.25, 10.15]
Porcaro 2015	19.2	9.21	108	16.5	9.88	43	1.6%	2.70 [-0.73, 6.13]
Tewari,2003	6.35	4.52	200	7.49	5.1	100	13.7%	-1.14 [-2.32, 0.04]
**Total (95% CI)**			**837**			**565**	**100.0%**	**1.61 [1.18, 2.05]**

Heterogeneity: Chi² = 115.32, df = 3 (P < 0.00001); I² = 97%
Test for overall effect: Z = 7.25 (P < 0.00001)

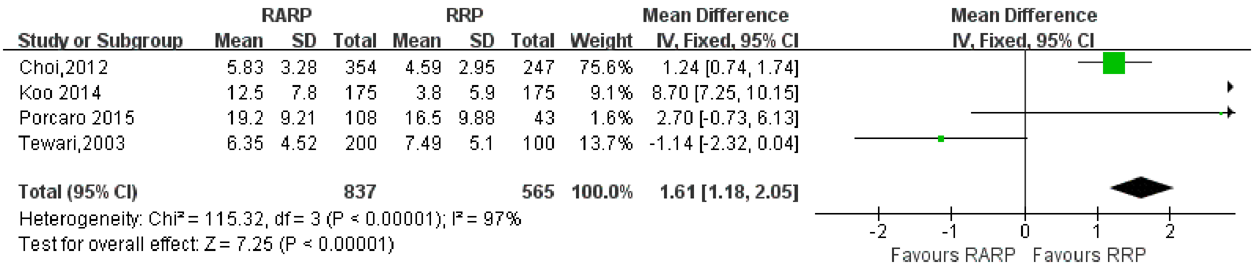

Fig.S9. Forest plot and meta-analysis of lymph node yield between RARP and RRP. RARP= robot-assisted radical prostatectomy; RRP= retropubic radical prostatectomy.

Study or Subgroup	RARP Events	RARP Total	RRP Events	RRP Total	Weight	Odds Ratio M-H, Fixed, 95% CI
Carlsson,2010	2	1253	8	485	32.4%	0.10 [0.02, 0.45]
Di Pierro,2011	0	75	1	75	4.2%	0.33 [0.01, 8.20]
Drouin,2009	0	71	1	83	3.9%	0.38 [0.02, 9.59]
Ficarra,2009	1	103	0	105	1.4%	3.09 [0.12, 76.68]
Menon,2002	0	30	1	30	4.2%	0.32 [0.01, 8.24]
OU,2009	0	30	1	30	4.2%	0.32 [0.01, 8.24]
Sugihara,2014	0	2126	34	7202	44.3%	0.05 [0.00, 0.80]
Tewari,2003	0	200	1	100	5.6%	0.17 [0.01, 4.10]
**Total (95% CI)**		**3888**		**8110**	**100.0%**	**0.16 [0.07, 0.39]**
Total events	3		47			

Heterogeneity: Chi² = 5.22, df = 7 (P = 0.63); I² = 0%
Test for overall effect: Z = 4.03 (P < 0.0001)

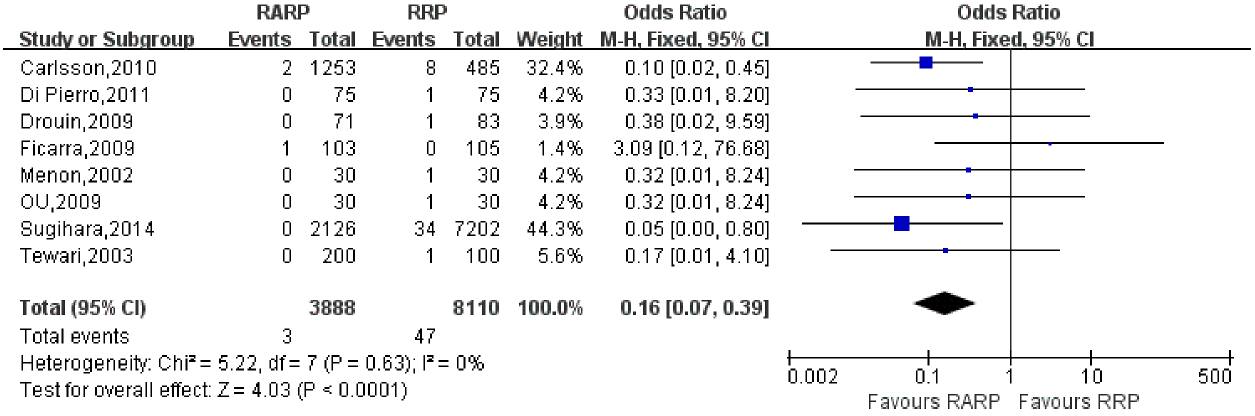

Fig.S10. Forest plot and meta-analysis of rectal injury between RARP and RRP. RARP= robot-assisted radical prostatectomy; RRP= retropubic radical prostatectomy.

Study or Subgroup	RARP Events	RARP Total	RRP Events	RRP Total	Weight	Odds Ratio M-H, Fixed, 95% CI
Ahlering,2004	1	60	0	60	0.3%	3.05 [0.12, 76.39]
Carlsson,2010	2	1253	5	485	3.9%	0.15 [0.03, 0.79]
Davis 2014	106	27348	107	13840	75.8%	0.50 [0.38, 0.65]
Doumerc,2010	0	212	1	502	0.5%	0.79 [0.03, 19.39]
Drouin,2009	0	71	1	83	0.7%	0.38 [0.02, 9.59]
Krambeck,2008	1	284	9	588	3.1%	0.23 [0.03, 1.80]
Pilecki,2014	20	4374	10	1097	8.5%	0.50 [0.23, 1.07]
Sugihara,2014	1	2126	14	7202	3.4%	0.24 [0.03, 1.84]
Wallerstedt 2015	3	1847	5	778	3.8%	0.25 [0.06, 1.06]
**Total (95% CI)**		**37575**		**24635**	**100.0%**	**0.47 [0.37, 0.59]**
Total events	134		152			

Heterogeneity: Chi² = 5.04, df = 8 (P = 0.75); I² = 0%
Test for overall effect: Z = 6.23 (P < 0.00001)

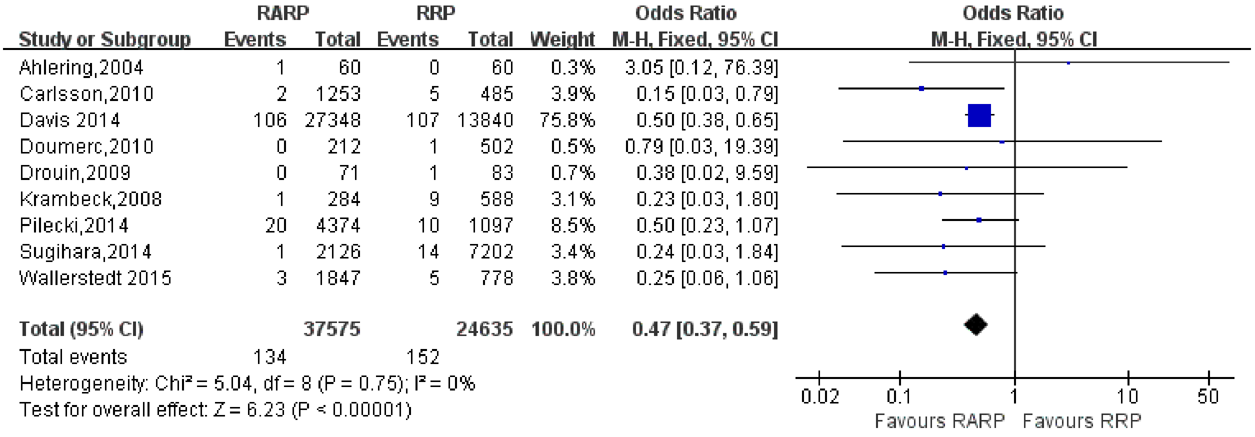

Fig.S11. Forest plot and meta-analysis of pulmonary embolism between RARP and RRP. RARP= robot-assisted radical prostatectomy; RRP= retropubic radical prostatectomy.

Study or Subgroup	RARP Events	RARP Total	RRP Events	RRP Total	Weight	Odds Ratio M-H, Random, 95% CI
Carlsson,2010	6	1253	29	485	15.1%	0.08 [0.03, 0.18]
Di Pierro,2011	0	75	2	75	4.2%	0.19 [0.01, 4.12]
Ficarra,2009	0	103	1	105	3.9%	0.34 [0.01, 8.36]
Menon,2002	3	30	3	30	9.3%	1.00 [0.19, 5.40]
Nelson,2007	2	629	5	374	9.6%	0.24 [0.05, 1.22]
Pilecki,2014	32	4374	34	1097	18.1%	0.23 [0.14, 0.38]
Ryu,2013	1	524	14	341	7.5%	0.04 [0.01, 0.34]
Sugihara,2014	1	2126	68	7202	7.8%	0.05 [0.01, 0.36]
Tewari,2003	2	200	1	100	6.0%	1.00 [0.09, 11.16]
Wallerstedt 2015	59	1847	42	778	18.6%	0.58 [0.39, 0.87]
**Total (95% CI)**		**11161**		**10587**	**100.0%**	**0.23 [0.11, 0.46]**
Total events	106		199			

Heterogeneity: Tau² = 0.66; Chi² = 31.49, df = 9 (P = 0.0002); I² = 71%
Test for overall effect: Z = 4.08 (P < 0.0001)

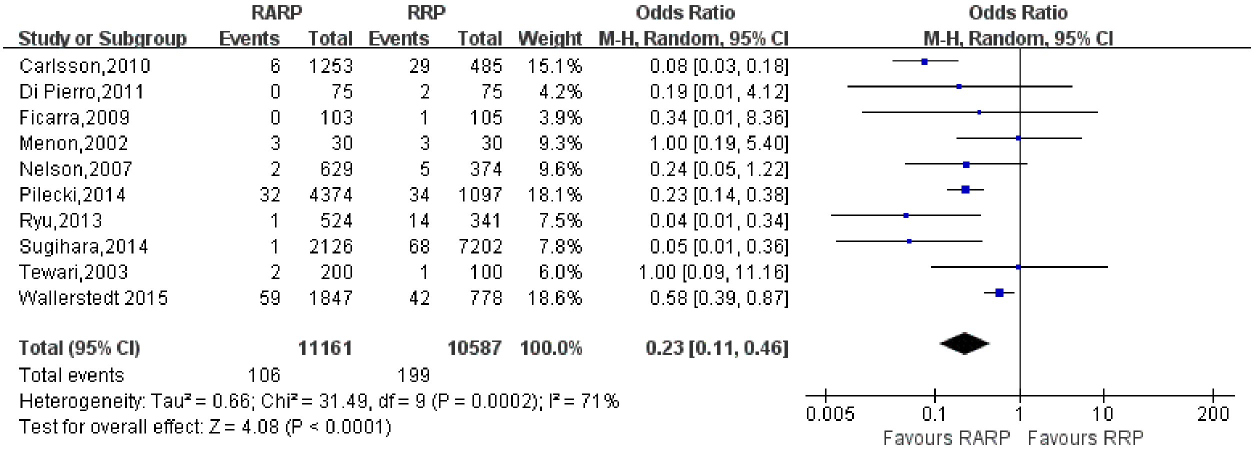

Fig.S12. Forest plot and meta-analysis of wound infections between RARP and RRP. RARP= robot-assisted radical prostatectomy; RRP= retropubic radical prostatectomy.

Study or Subgroup	RARP Events	RARP Total	RRP Events	RRP Total	Weight	Odds Ratio M-H, Random, 95% CI
Breyer,2010	4	293	18	695	27.2%	0.52 [0.17, 1.55]
Carlsson,2010	3	1253	22	485	25.4%	0.05 [0.02, 0.17]
Krambeck,2008	3	294	24	588	25.4%	0.24 [0.07, 0.81]
Yi,2010	2	153	25	641	22.0%	0.33 [0.08, 1.39]
**Total (95% CI)**		**1993**		**2409**	**100.0%**	**0.21 [0.08, 0.60]**
Total events	12		89			

Heterogeneity: Tau² = 0.71; Chi² = 8.39, df = 3 (P = 0.04); I² = 64%
Test for overall effect: Z = 2.93 (P = 0.003)

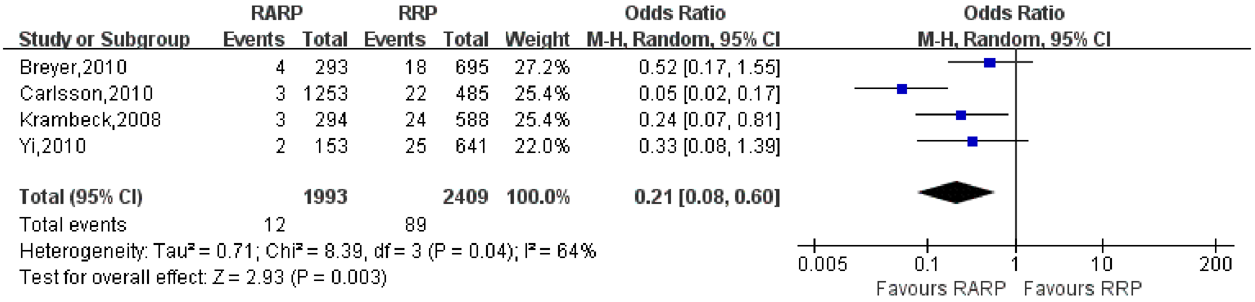

Fig.S13. Forest plot and meta-analysis of bladder neck contracture between RARP and RRP. RARP= robot-assisted radical prostatectomy; RRP= retropubic radical prostatectomy.

Study or Subgroup	RARP Events	RARP Total	RRP Events	RRP Total	Weight	Odds Ratio M-H, Fixed, 95% CI
Menon,2002	1	30	1	30	0.9%	1.00 [0.06, 16.76]
Wallerstedt 2015	100	1847	58	778	71.3%	0.71 [0.51, 0.99]
Wood,2007	16	165	32	152	27.8%	0.40 [0.21, 0.77]
**Total (95% CI)**		**2042**		**960**	**100.0%**	**0.63 [0.47, 0.84]**
Total events	117		91			

Heterogeneity: Chi² = 2.44, df = 2 (P = 0.29); I² = 18%
Test for overall effect: Z = 3.13 (P = 0.002)

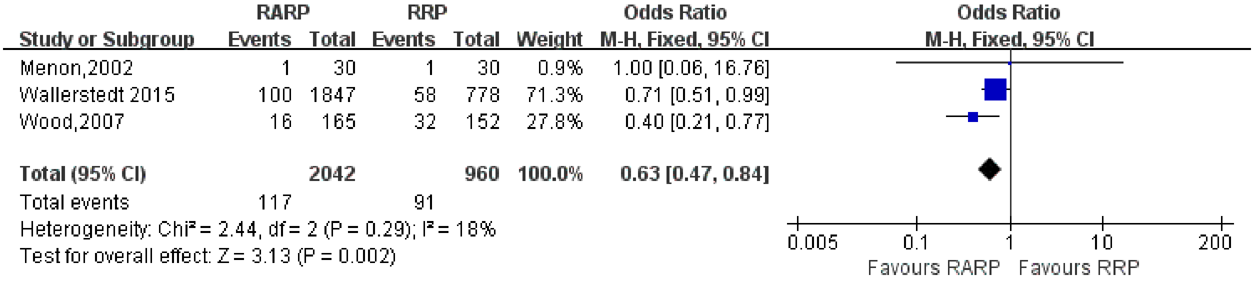

Fig.S14. Forest plot and meta-analysis of urinary retention between RARP and RRP. RARP= robot-assisted radical prostatectomy; RRP= retropubic radical prostatectomy.

	RARP		RRP			Odds Ratio
**Study or Subgroup**	**Events**	**Total**	**Events**	**Total**	**Weight**	**M-H, Fixed, 95% CI**
Ahlering,2004	0	60	3	60	7.2%	0.14 [0.01, 2.69]
Di Pierro,2011	1	75	0	75	1.0%	3.04 [0.12, 75.83]
Krambeck,2008	2	294	13	588	17.9%	0.30 [0.07, 1.35]
Nelson,2007	3	629	4	374	10.4%	0.44 [0.10, 1.99]
Pilecki,2014	24	4374	6	1097	19.8%	1.00 [0.41, 2.46]
Tewari,2003	1	200	1	100	2.8%	0.50 [0.03, 8.04]
Wallerstedt 2015	4	1847	14	778	40.9%	0.12 [0.04, 0.36]
**Total (95% CI)**		**7479**		**3072**	**100.0%**	**0.40 [0.25, 0.66]**
Total events	35		41			
Heterogeneity: Chi² = 10.82, df = 6 (P = 0.09); I² = 45%						
Test for overall effect: Z = 3.63 (P = 0.0003)						

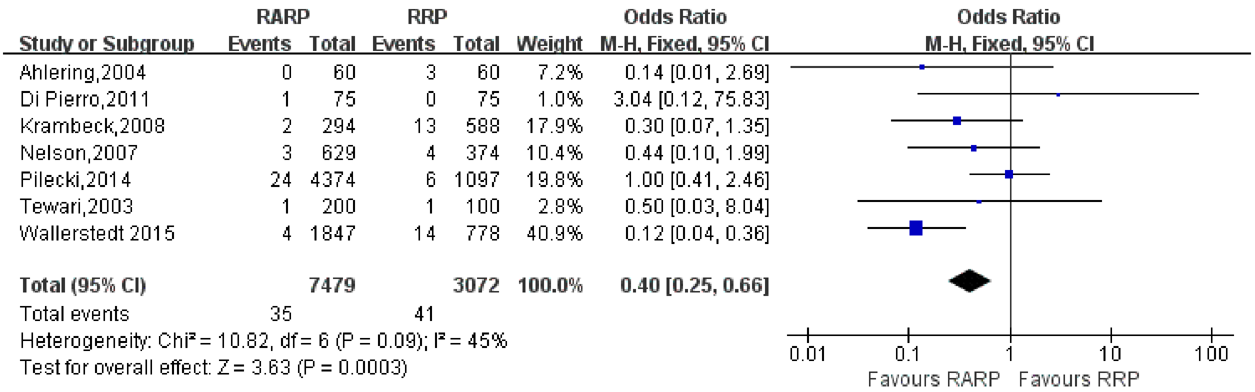

Fig.S15. Forest plot and meta-analysis of deep venous thrombosis between RARP and RRP. RARP= robot-assisted radical prostatectomy; RRP= retropubic radical prostatectomy.

	RARP		RRP			Odds Ratio
**Study or Subgroup**	**Events**	**Total**	**Events**	**Total**	**Weight**	**M-H, Fixed, 95% CI**
Ahlering,2004	1	60	0	60	0.1%	3.05 [0.12, 76.39]
Carlsson,2010	13	1253	8	485	1.3%	0.63 [0.26, 1.52]
D'Alonzo,2009	1	256	0	280	0.1%	3.29 [0.13, 81.21]
Davis 2014	870	27348	686	13840	97.4%	0.63 [0.57, 0.70]
Di Pierro,2011	1	75	7	75	0.8%	0.13 [0.02, 1.09]
Drouin,2009	0	71	1	83	0.2%	0.38 [0.02, 9.59]
OU,2009	1	30	2	30	0.2%	0.48 [0.04, 5.63]
Wallerstedt 2015	14	1847	1	778	0.2%	5.93 [0.78, 45.21]
**Total (95% CI)**		**30940**		**15631**	**100.0%**	**0.64 [0.58, 0.70]**
Total events	901		705			
Heterogeneity: Chi² = 8.87, df = 7 (P = 0.26); I² = 21%						
Test for overall effect: Z = 8.80 (P < 0.00001)						

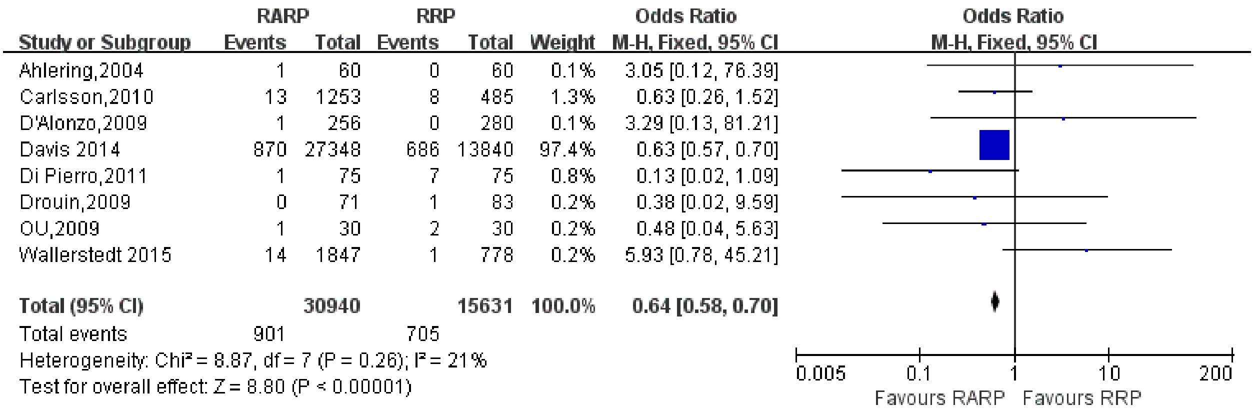

Fig.S16. Forest plot and meta-analysis of urinary leakage between RARP and RRP. RARP= robot-assisted radical prostatectomy; RRP= retropubic radical prostatectomy.

	RARP		RRP			Odds Ratio
**Study or Subgroup**	**Events**	**Total**	**Events**	**Total**	**Weight**	**M-H, Fixed, 95% CI**
Carlsson,2010	1	1253	4	485	18.4%	0.10 [0.01, 0.86]
Di Pierro,2011	4	75	7	75	21.1%	0.55 [0.15, 1.95]
Drouin,2009	0	71	1	83	4.4%	0.38 [0.02, 9.59]
Krambeck,2008	2	294	9	588	19.0%	0.44 [0.09, 2.05]
Nelson,2007	2	629	1	374	4.0%	1.19 [0.11, 13.17]
OU,2009	1	30	0	30	1.5%	3.10 [0.12, 79.23]
Silberstein,2012	4	126	1	126	3.1%	4.10 [0.45, 37.19]
Tewari,2003	0	200	2	100	10.6%	0.10 [0.00, 2.07]
Wallerstedt 2015	3	1847	4	778	17.9%	0.31 [0.07, 1.41]
**Total (95% CI)**		**4525**		**2639**	**100.0%**	**0.52 [0.29, 0.94]**
Total events	17		29			
Heterogeneity: Chi² = 8.93, df = 8 (P = 0.35); I² = 10%						
Test for overall effect: Z = 2.16 (P = 0.03)						

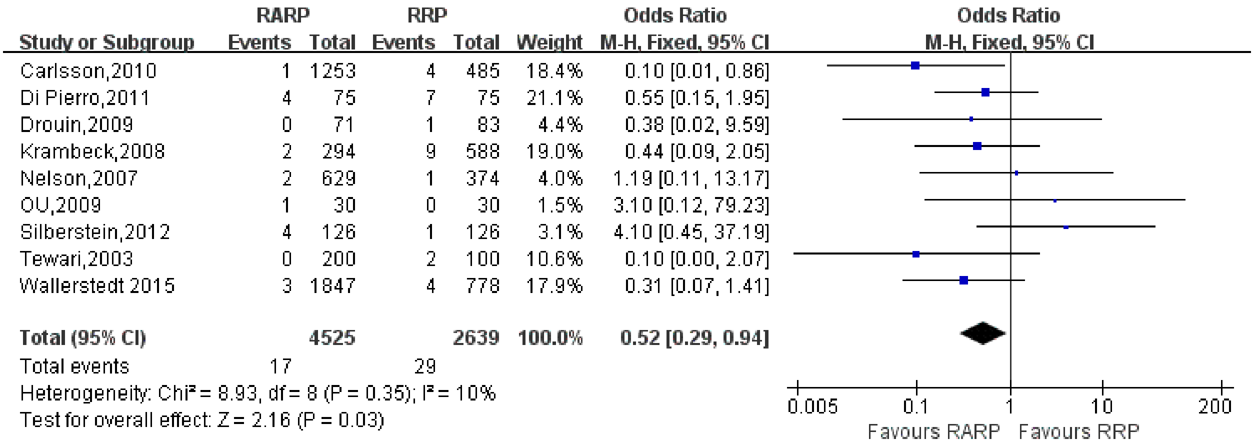

Fig.S17. Forest plot and meta-analysis of lymphocele between RARP and RRP. RARP= robot-

assisted radical prostatectomy; RRP= retropubic radical prostatectomy.

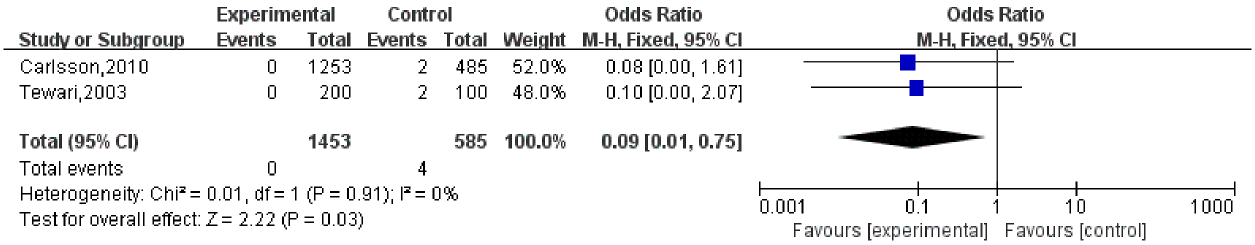

Fig.S18. Forest plot and meta-analysis of obturator never injury between RARP and RRP. RARP= robot-assisted radical prostatectomy; RRP= retropubic radical prostatectomy.

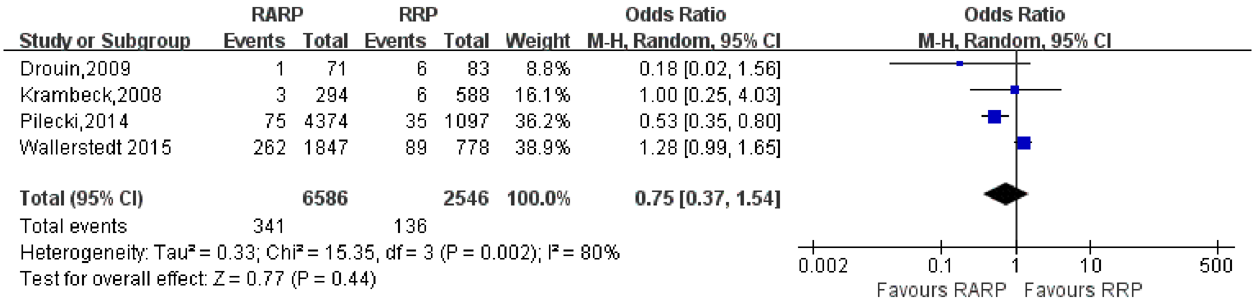

Fig.S19. Forest plot and meta-analysis of UTI between RARP and RRP. RARP= robot-assisted radical prostatectomy; RRP= retropubic radical prostatectomy.

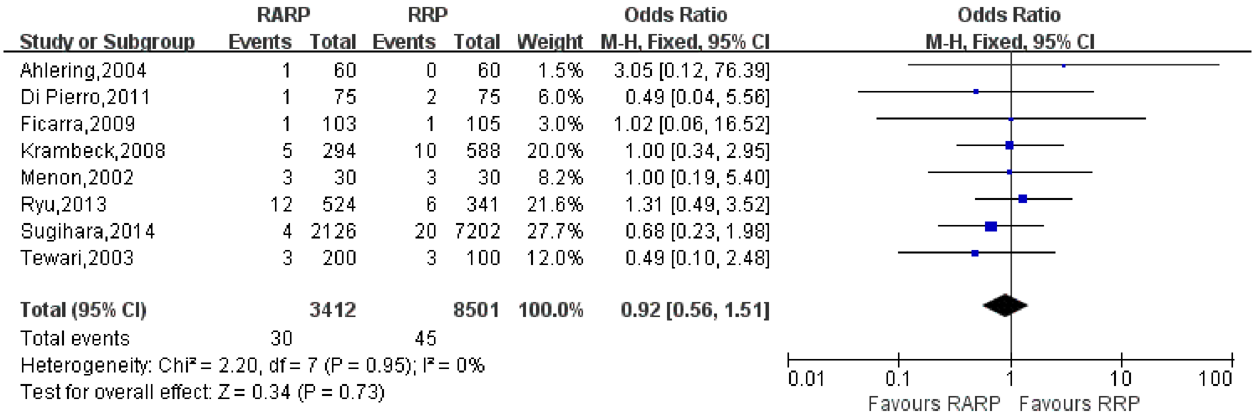

Fig.S20. Forest plot and meta-analysis of ileus between RARP and RRP. RARP= robot-assisted radical prostatectomy; RRP= retropubic radical prostatectomy.

Study or Subgroup	RARP Events	RARP Total	RRP Events	RRP Total	Weight	Odds Ratio M-H, Fixed, 95% CI
Choo,2013	74	77	165	176	2.3%	1.64 [0.45, 6.07]
Di Pierro,2011	40	45	60	75	2.9%	2.00 [0.67, 5.94]
Ficarra,2009	100	103	92	105	1.5%	4.71 [1.30, 17.06]
Gagnon 2014	145	152	138	145	3.8%	1.05 [0.36, 3.07]
Krambeck,2008	270	294	551	588	17.3%	0.76 [0.44, 1.29]
Ong 2015	124	639	162	731	70.3%	0.85 [0.65, 1.10]
OU,2009	30	30	29	30	0.3%	3.10 [0.12, 79.23]
Rocco,2009	77	79	191	217	1.5%	5.24 [1.21, 22.62]
Son,2013	146	146	110	112	0.2%	6.63 [0.32, 139.47]
**Total (95% CI)**		**1565**		**2179**	**100.0%**	**1.03 [0.84, 1.27]**
Total events	1006		1498			

Heterogeneity: Chi² = 17.41, df = 8 (P = 0.03); I² = 54%

Test for overall effect: Z = 0.31 (P = 0.75)

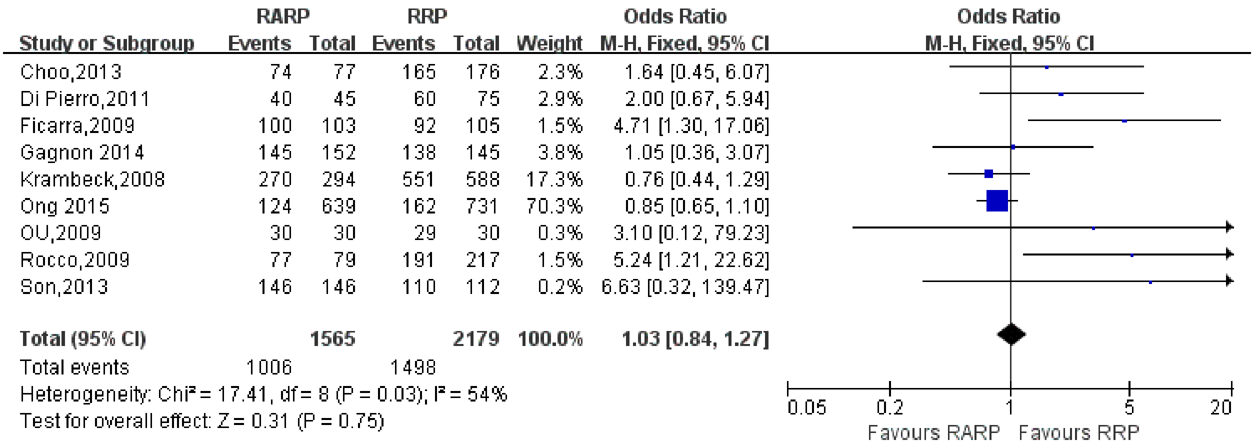

Fig.S21. Forest plot and meta-analysis of 12-mo urinary continence rate between RARP and RRP. RARP= robot-assisted radical prostatectomy; RRP= retropubic radical prostatectomy.
